# Crystal structures of the (η^2^:η^2^-cyclo­octa-1,5-diene)(η^6^-toluene)­iridium(I) cation and μ-chlorido-iridium(III) complexes of 2-(phosphinito)- and 2-(phosphinometh­yl)anthra­quinone ligands

**DOI:** 10.1107/S2056989024008922

**Published:** 2024-09-30

**Authors:** Sachin Thackeray, James Mahoney, Ashleigh Arrington, Miles Wilklow-Marnell, William W. Brennessel

**Affiliations:** a Department of Chemistry State University of New York at New Paltz New Paltz NY 12561 USA; b Department of Chemistry 120 Trustee Road University of Rochester,Rochester NY 14627 USA; Texas A & M University, USA

**Keywords:** crystal structure, pincer ligands, phosphine ligands, iridium, quinone, chlorido-bridged, hydrido complex, metallacycle

## Abstract

When reacted in dry, degassed toluene, [Ir(COD)Cl]_2_ (COD = cyclo­octa-1,5-diene) and 2 equivalents of 2-(di-*tert*-butyl­phosphinito)anthra­quinone (^tBu^POAQH) were found to form a unique tri-iridium compound consisting of one monoanionic dinuclear tri-μ-chlorido complex bearing one bidentate ^tBu^POAQ ligand per iridium, which was charge-balanced by an outer sphere [Ir(toluene)(COD)]^+^ ion, the structure of which has not previously been reported. This product, which is a toluene solvate, namely, (η^2^:η^2^-cyclo­octa-1,5-diene)(η^6^-toluene)­iridium(I) tri-μ-chlorido-bis­({3-[(di-*tert*-butyl­phosphan­yl)­oxy]-9,10-dioxoanthracen-2-yl}hydridoiridium(III)) toluene monosolvate, [Ir(C_7_H_8_)(C_8_H_12_)][Ir_2_H_2_(C_22_H_24_O_3_P)_2_Cl_3_]·C_7_H_8_ or [Ir(toluene)(COD)][Ir(κ-*P*,*C*-^tBu^POAQ)(H)]_2_(μ-Cl)_3_]·toluene, formed as small orange platelets at room temperature, crystallizing in the triclinic space group *P*. The cation and anion are linked *via* weak C—H⋯O inter­actions. The stronger inter­molecular attractions are likely the offset parallel π–π inter­actions, which occur between the toluene ligands of pairs of inverted cations and between pairs of inverted anthra­quinone moieties, the latter of which are capped by toluene solvate mol­ecules, making for π-stacks of four mol­ecules each. The related ligand, 2-(di-*tert*-butyl­phosphinometh­yl)-anthra­quinone (^tBu^PCAQH), did not form crystals suitable for X-ray diffraction under analogous reaction conditions. However, when the reaction was conducted in chloro­form, yellow needles readily formed following addition of 1 atm of carbon monoxide. Diffraction studies revealed a neutral, dinuclear, di-μ-chlorido complex, di-μ-chlorido-bis­(carbon­yl{3-[(di-*tert*-butyl­phosphan­yl)­oxy]-9,10-dioxoanthracen-2-yl}hydridoiridium(I)), [Ir_2_H_2_(C_23_H_26_O_2_P)_2_Cl_2_(CO)_2_] or [Ir(κ-*P*,*C*-^tBu^PCAQ)(H)(CO)(μ-Cl)]_2_, Ir_2_C_48_H_54_Cl_2_O_6_P_2_, again crystallizing in space group *P*. Offset parallel π–π inter­actions between anthra­quinone groups of adjacent mol­ecules link the mol­ecules in one dimension.

## Abstract 2

Phosphinito and phosphinomethyl ligands incorporating an anthra­quinone moiety were reacted with bis­(cyclo­octa-1,5-diene)diiridium(I) dichloride to afford novel diiridium species. Intended as pincer-type tridentate ligands, bidentate binding modes were determined by X-ray crystallography. The anionic μ-tri­chlorido phosphinito complex formed is charged-balanced by one [Ir(toluene)(cyclo­octa-1,5-diene)]^+^ per asymmetric unit, the structure of which has not previously been reported despite a long history of use as an Ir^I^ source in organometallic chemistry.

## 1. Chemical context

**Figure 1:**
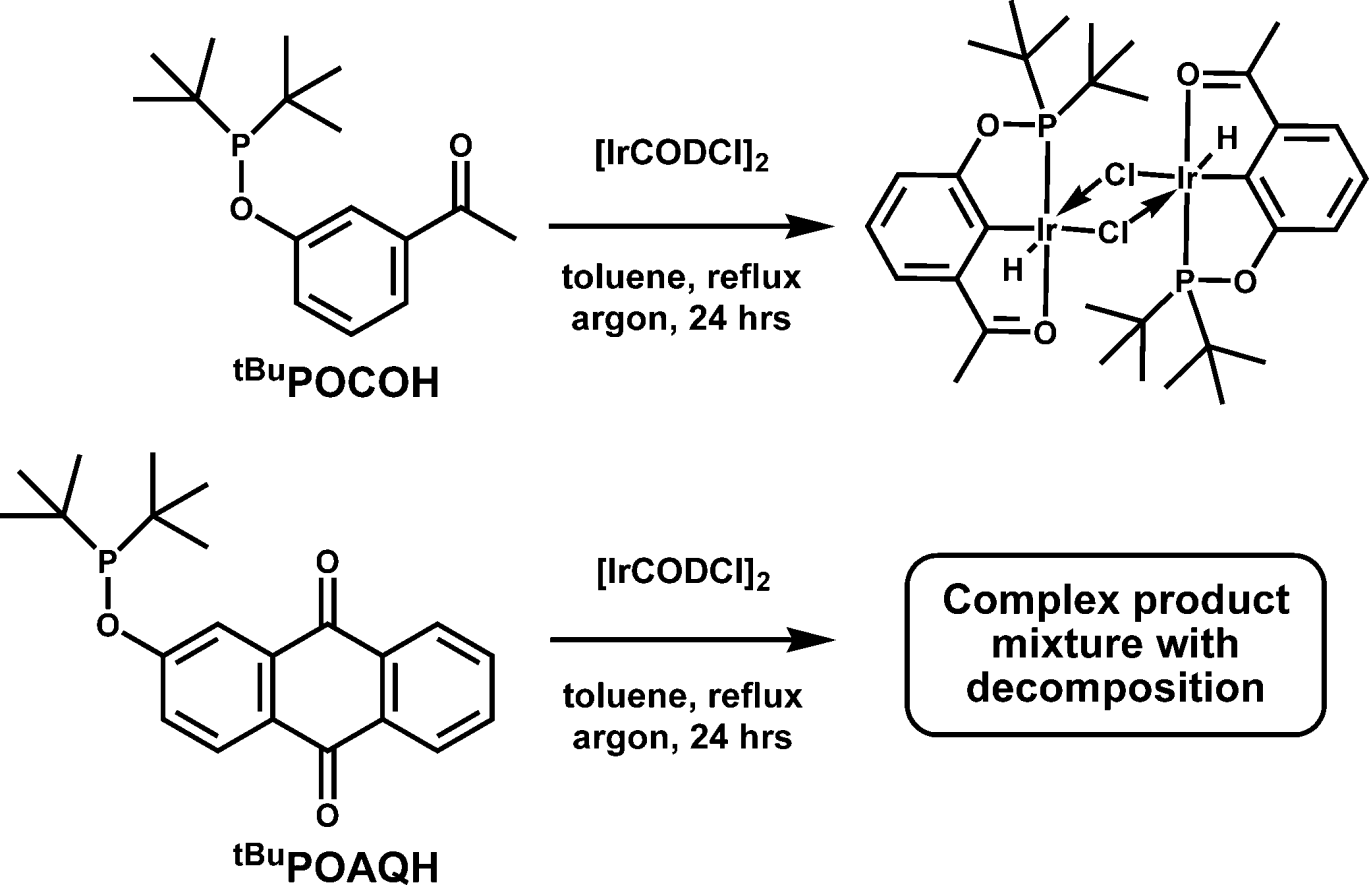
Successful metalation of ^tBu^POCOH using [Ir(COD)Cl]_2_ and attempted metalation of ^tBu^POAQH.

Tridentate, meridional ligands known as ‘pincers’ have become ubiquitous in organometallic chemistry, particularly in complexes of platinum group metals (Albrecht & van Koten, 2001 ▸), though many systems incorporating first-row transition metals now exist (Morales-Morales, 2018 ▸; Alig *et al.*, 2019 ▸). Complexes of iridium have held a central role in the development of pincer chemistry since the first known reports of organometallic pincer complexes (Moulton & Shaw, 1976 ▸), and are probably most notable for advances in homogeneous C—H activation chemistry and alkane de­hydrogenation (Choi *et al.*, 2011 ▸). Pincer ligands have found widespread use due to their tunability, their ability to enforce reactive conformations, and the enhanced stability of these systems (van der Boom *et al.*, 2003 ▸; Roddick, 2013 ▸). The enhanced stability of pincer complexes largely stems from their tridentate binding mode; however, ligands intended as pincer-type do not always bind in a tridentate fashion. Sometimes bidentate, or even metal bridging, binding modes are encountered. Pincers bearing two phosphinite groups, known as POCOP ligands, have been particularly well studied (Morales-Morales, 2008 ▸). Previously, we reported a modification of this common framework bearing one phosphinite and one ketone group, 3-(di-*tert*-butyl­phosphinito)aceto­phenone, or ^tBu^POCOH (Wilklow-Marnell & Brennessel, 2019 ▸). When refluxed in toluene for 24 h with 0.5 molar equivalents of [Ir(COD)Cl]_2_ (COD = cyclo­octa-1,5-diene), the desired pincer-ligated iridium species was obtained in good yield (Fig. 1 ▸). However, when metalation of the related ligand, 2-(di-*tert*-butyl­phosphinito)anthra­quinone (^tBu^POAQH), was conducted under identical conditions, a mixture of unidentified products was obtained which resisted efforts to separate cleanly (Fig. 1 ▸).
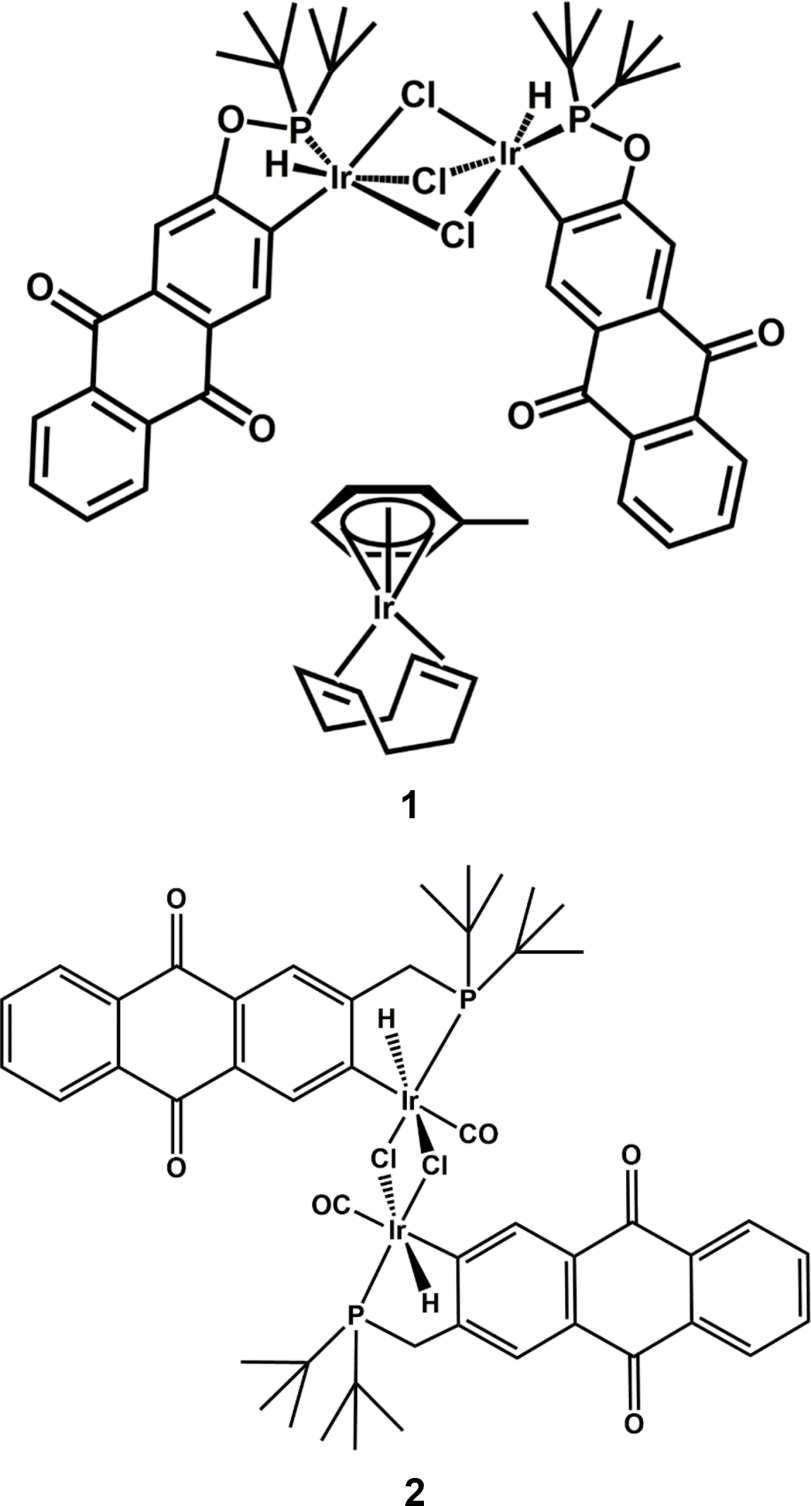


**Figure 2:**
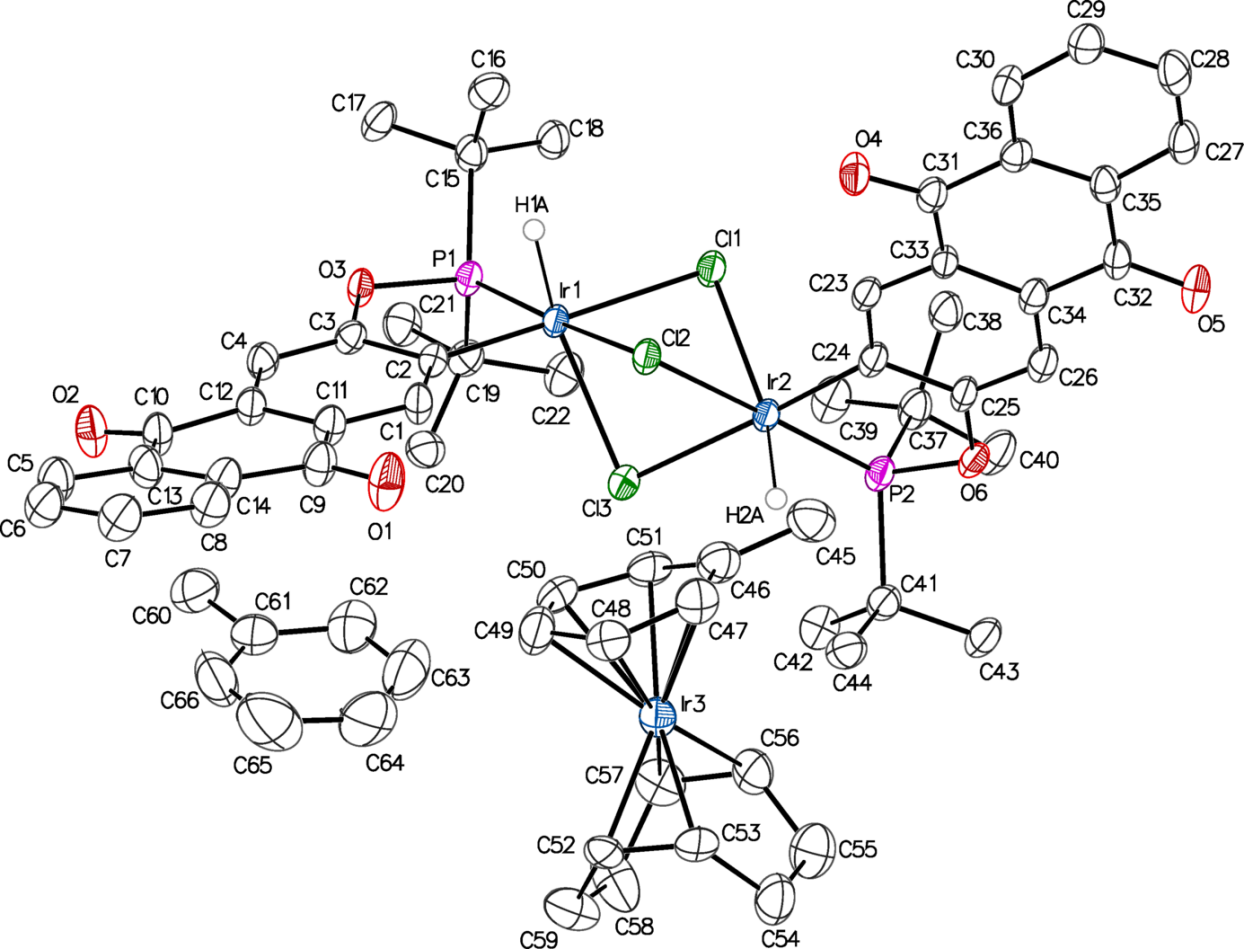
Anisotropic displacement ellipsoid plot of **1** drawn at the 50% probability level with all H atoms omitted except for hydrido ligands. The minor component of disorder is not shown.

Before any heating, and almost immediately upon mixing, a significant color change from orange to dark reddish brown was noted when ^tBu^POAQH was reacted with [Ir(COD)Cl]_2_ in toluene at room temperature, indicating that some degree of ligation of ^tBu^POAQH had occurred. In light of the failure of forming a pincer complex when refluxed in toluene, the reaction was again attempted but allowed to remain at room temperature. The ^31^P NMR spectrum indicated mainly a single product had formed with a resonance at 161 ppm in toluene and some free ^tBu^POAQH remaining. Over a period of several days, a fine orange crystalline material separated from the solution, which was determined by single crystal structure determination to be the unique complex [Ir(η^6^-toluene)(η^2^:η^2^-COD)] [(^tBu^POAQIrH)_2_(μ-Cl)_3_]·toluene (**1**) as shown in Fig. 2 ▸. The formation and isolation of [Ir(toluene)(COD)]^+^ and other [Ir(arene)(COD)]^+^ complexes have been previously demonstrated (Sievert & Muetterties, 1981 ▸; Kanchiku *et al.*, 2007 ▸); however, a structure containing the [Ir(toluene)(COD)]^+^ cation has not yet been reported to date.

It was considered that under reflux conditions trace moisture may have led to hydrolysis of the ligand P—O bond, and the carbon analog, 2-(di-*tert*-butyl­phosphinometh­yl)anthra­quinone (^tBu^PCAQH), was synthesized in hopes it might resist this. However, metalation of ^tBu^PCAQH with [Ir(COD)Cl]_2_ in refluxing toluene again failed to produce an isolable pincer-ligated product. When allowed to react and remain at room temperature, a solid material did eventually separate from solution; however, only polycrystalline material or crystals too small for diffraction were obtained.

**Figure 3:**
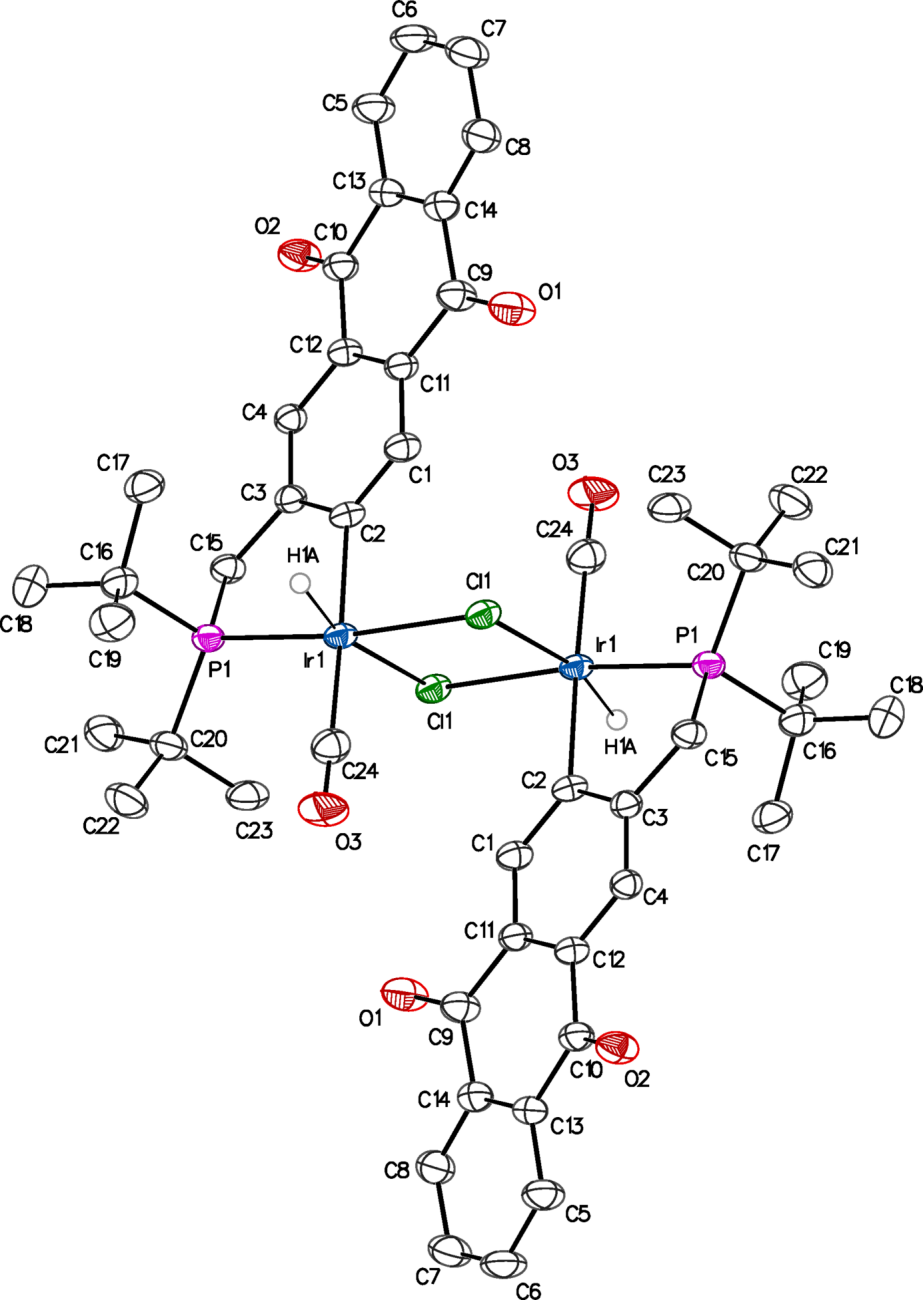
Anisotropic displacement ellipsoid plot of **2** drawn at the 50% probability level with all H atoms omitted except for hydrido ligands. The symmetry-equivalent portion of the mol­ecule was generated by the inversion operation 1 − *x*, 1 − *y*, 1 − *z*.

When formed in toluene or chloro­form, isolated and removed of volatiles under vacuum, then redissolved in chloro­form (or deutero­chloro­form) and exposed to an atmosphere of carbon monoxide, a change of color to pale yellow was noted. Crystals formed over a 16 h period. Single crystal X-ray diffraction revealed this product to be the charge neutral di-iridium complex, [Ir(*κ*-*P*,*C*-PCAQ)H(CO)(μ-Cl)]_2_ (**2**), with the intended pincer ligand again binding instead in a bidentate manner (Fig. 3 ▸). In this report, we provide the syntheses of both ^tBu^POAQH and ^tBu^PCAQH ligands, the isolation of complexes **1** and **2**, and compare their structures to related iridium complexes.

## 2. Structural commentary

Single-crystal X-ray diffraction analysis determined the structure of **1** to be a tri-iridium species with two iridium-containing complex ions as a toluene solvate (Fig. 2 ▸). The asymmetric unit contains one monocationic iridium complex, one monoanionic di-iridium complex, and one toluene solvent mol­ecule of crystallization, all in general positions in space group *P*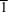. The anionic complex (**1an**) consists of two iridi­um(III) centers, each ligated by one bidentate ^tBu^POAQ ligand *via* P and C atoms, one hydrido ligand (resulting from C—H activation of ^tBu^POAQH), and three bridging chlorido ligands. The geometry at each iridium center is distorted octa­hedral (Stiefel & Brown, 1972 ▸). The cationic complex (**1cat**) consists of an iridium(I) center bound η^6^ to a toluene ligand and bound in a bis-η^2^ manner to unconjugated diene COD, giving a geometry akin to a ‘planar’ two-legged piano stool complex (Ward *et al.*, 1997 ▸), wherein the two legs are the midpoints of the double bonds of the COD ligand.

The majority, if not all, six-coordinate Ir^III^ complexes adopt octa­hedral geometries. In fact, as a mol­ecular complex, there has yet to be a report of a trigonal–prismatic hexa­coordinate iridium species to our knowledge (Yellowlees & Macnamara, 2003 ▸; Cremades *et al.*, 2010 ▸). The slight distortion from an octa­hedral arrangement seen in **1an** likely results from the steric bulk and restricted bite angle of the ^tBu^POAQ ligand, as well as the limited separation of the three bridging chlorides which, as an IrCl_3_ unit, act as a tridentate ligand for the other iridium in the complex. Octa­hedral complexes are known to distort by undergoing trigonal twisting into a metaprismatic geometry somewhere between octa­hedral and trigonal prismatic forms when chelating ligands with rigid *L*—*L* distances are present (Cremades *et al.*, 2010 ▸; Alvarez, 2015 ▸). This occurs because most five-membered chelate rings display bite angles of < 90° (Aguilà *et al.*, 2009 ▸), which better suit the ideal bond angle between ligands in a trigonal–prismatic geometry of 81.8°, as opposed to 90° for octa­hedral. However, the bite angle alone can only rarely induce a true trigonal–prismatic geometry.

In the case of the **1an**, the Ir—Cl bond lengths are not uniform, but the bonds *trans* to the hydrido ligands are much longer by comparison [2.5819 (11) Å *versus* 2.4782 (11) and 2.4818 (11) Å for Ir1; 2.5476 (11) *versus* 2.4963 (11) and 2.4780 (12) Å for Ir2; Table 1 ▸], fitting with the strong *trans*-influence of a hydrido ligand. The Cl—Ir—Cl bond angles have a range of 78.35 (4)–82.68 (4)°, and average to 80.38 (7)° at Ir1 and 80.64 (7)° at Ir2, quite close to the ideal angles of a trigonal–prismatic geometry [81.8°]. Ostensibly, the steric influence of *tert*-butyl groups from the ^tBu^POAQ ligand on this are evident as the P—Ir—Cl bond angles for the two chlorides *cis* to P are rather large at 107.33 (4) and 101.24 (4)° for Ir1 and 106.79 (4) and 103.93 (4)° for Ir2, while the remaining bond angles at the metal centers do not deviate far from the ideal value of 90° for an octa­hedral complex. However, for related Ir—(μ-Cl)_3_—Ir containing species with a range of ligand electronics/sterics, average Cl—Ir—Cl bond angles of roughly 79 to 82° are reported, indicating that the contraction of these angles is likely due mainly to the constraints of the IrCl_3_ fragment, as opposed to steric bulk of ^tBu^POAQ or an electronic preference for a metaprismatic geometry (Allevi *et al.*, 1998 ▸; Zhang *et al.*, 2004 ▸; Maekawa *et al.*, 2004*b* ▸; Dahlenburg *et al.*, 2008 ▸). The P—Ir—C angles of **1an** are appreciably constricted, averaging at 82.16 (19)°, due to being part of the five-membered chelate ring formed with ^tBu^POAQ. Related ^tBu^POCOIr and symmetric ^R^POCOPIr complexes for which structures have been determined display similar P—Ir—C bond angles, between approximately 78.8 to 81.9°, with larger angles associated with the less bulky mono-phosphinite POCO ligand, which can presumably approach closer to the metal. The Ir—P bond lengths of the reported structures (2.26 to 2.36 Å) are comparable to those of **1an** (avg. 2.19 (16) Å; Wilklow-Marnell *et al.*, 2019 ▸; Göttker-Schnetmann *et al.*, 2004 ▸; Goldberg *et al.*, 2015 ▸; Shafiei-Haghighi *et al.*, 2018 ▸). With all anthra­quinone C—O bond lengths of **1an** averaging 1.225 (13) Å, and average C—C distances of 1.397 (17) and 1.392 (10) Å for the proximal and distal aryl rings, respectively, bond lengths within the anthra­quinone moiety of **1an** are in close agreement with those of free anthra­quinone and representative 2,3-disubstituted anthra­quinones 2,3-di­chloro­anthra­quinone and 2-bromo-3-methyl­anthra­quinone, indicating little electronic disturbance of the ^tBu^POAQ aromatic system as would be expected for Ir^III^ metal centers (Ketker *et al.*, 1981 ▸; Lenstra & van Loock, 1984 ▸; Il’in *et al.*, 1975 ▸; Pascal *et al.*, 2017 ▸).

Though long known in the literature (Muetterties *et al.*, 1979 ▸, 1981 ▸), the structure of the [Ir(toluene)(COD)]^+^ cation (**1cat**) has yet to have been determined by diffraction studies, despite twenty other reported structures to date that contain an [Ir(η^6^-arene)(COD)]^+^ unit (see *Database survey*). The η^6^-toluene ligand is not quite planar (r.m.s. deviation of 0.066 Å). It is somewhat puckered toward iridium, with a fold along the C48⋯C51 vector: the angle between the C46–C48/C51 and C48—C51 planes is 11.2 (6)°. The Ir—C bond distances vary accordingly (Table 1 ▸).

The ring C C bond lengths of the toluene ligand and those of the coordinated ethyl­ene units of the COD ligand are indicative of significant backbonding from a low-valent Ir^I^ center into the ligand π^*^ orbitals and are consistent with bond lengths seen in other structures with [Ir(η^6^-arene)(COD)]^+^ or [Rh(η^6^-toluene)(COD)]^+^ cations (see *Database survey*). The average ring C—C bond length of free toluene is 1.38 Å [Cambridge Structural Database (CSD), version 5.45, November 2023; Groom *et al.*, 2016 ▸], while that of the ligand in **1cat** is elongated at 1.413 (19) Å. Likewise the average of the metal-coordinating C=C bonds of the COD ligand in **1cat** is 1.425 (13) Å, as compared to that of free COD (1.333 Å; Byrn *et al.*, 1990 ▸).

As determined by single-crystal X-ray diffraction, **2** was also found to contain a diiridium species (Fig, 3), but is neutral and is bridged by two chlorido ligands as opposed to three as in **1**. If **2** goes through an inter­mediate similar to **1** following ligation of ^tBu^PCAQH to iridium, then ostensibly, the third chloride has been displaced by coordination of one carbon monoxide (CO) ligand per iridium. Without the constraints of a third bridging chlorido ligand, the geometry at iridium adopts a somewhat more idealized octa­hedral geometry, though still distorted by the steric demands of the ^tBu^PCAQ ligand and remaining four-membered ring of the Ir_2_(μ-Cl)_2_ unit (Table 2 ▸). As expected, the C2—Ir—P1 bond angle is contracted due to being part of a metallacycle to 81.83 (10)°, very similar to the average angle of 82.3° seen in the related structure of [Ir(η^2^:η^2^-COD)]_2_ {η^6^-[κ^4^-C_6_H_2_(CH_2_P(*t*Bu_2_)_2_]Ir_2_H_2_Cl_3_}_2_ (Zhang *et al.*, 2004 ▸). In both structures, the fused-ring parts are not quite planar, with angles between the proximal and distal rings of 12.0 (4) and 7.9 (3)° in **1**, and 14.23 (15)° in **2**.

## 3. Supra­molecular features

**Figure 4:**
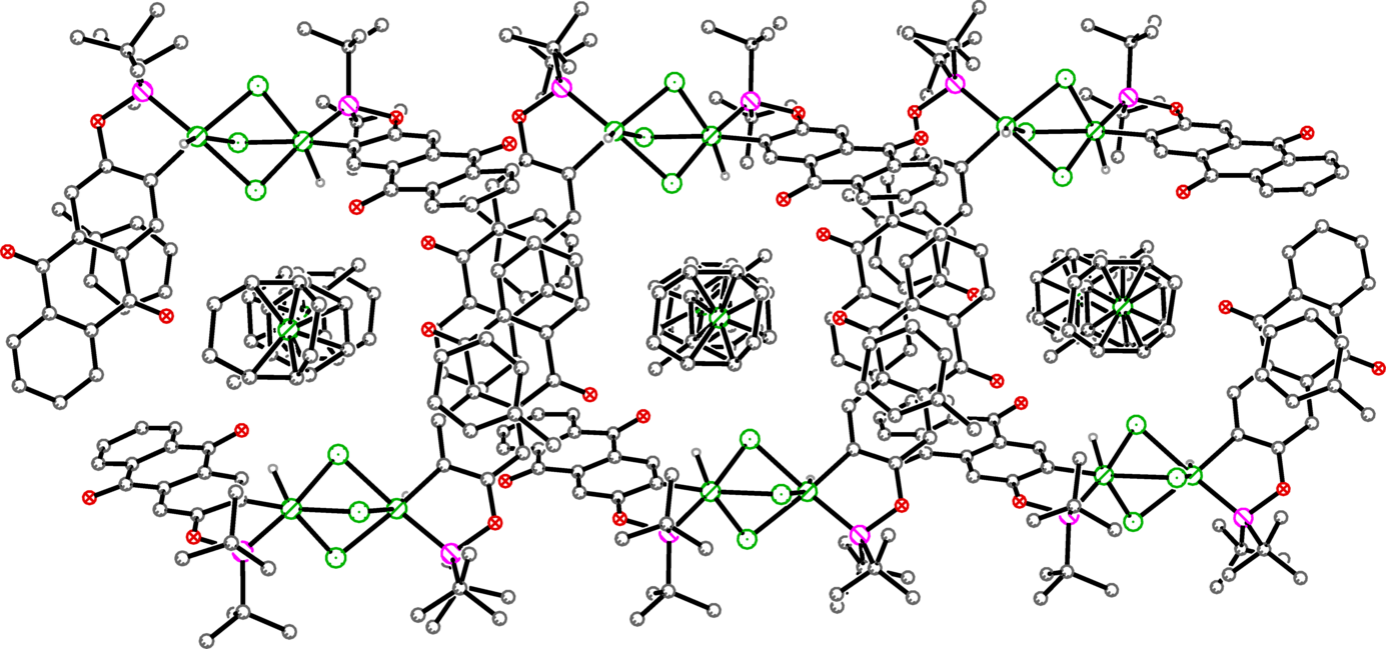
Packing plot of **1**. The inter­locked pattern (*via* π–π inter­actions) continues infinitely to the left and right.

**Figure 5:**
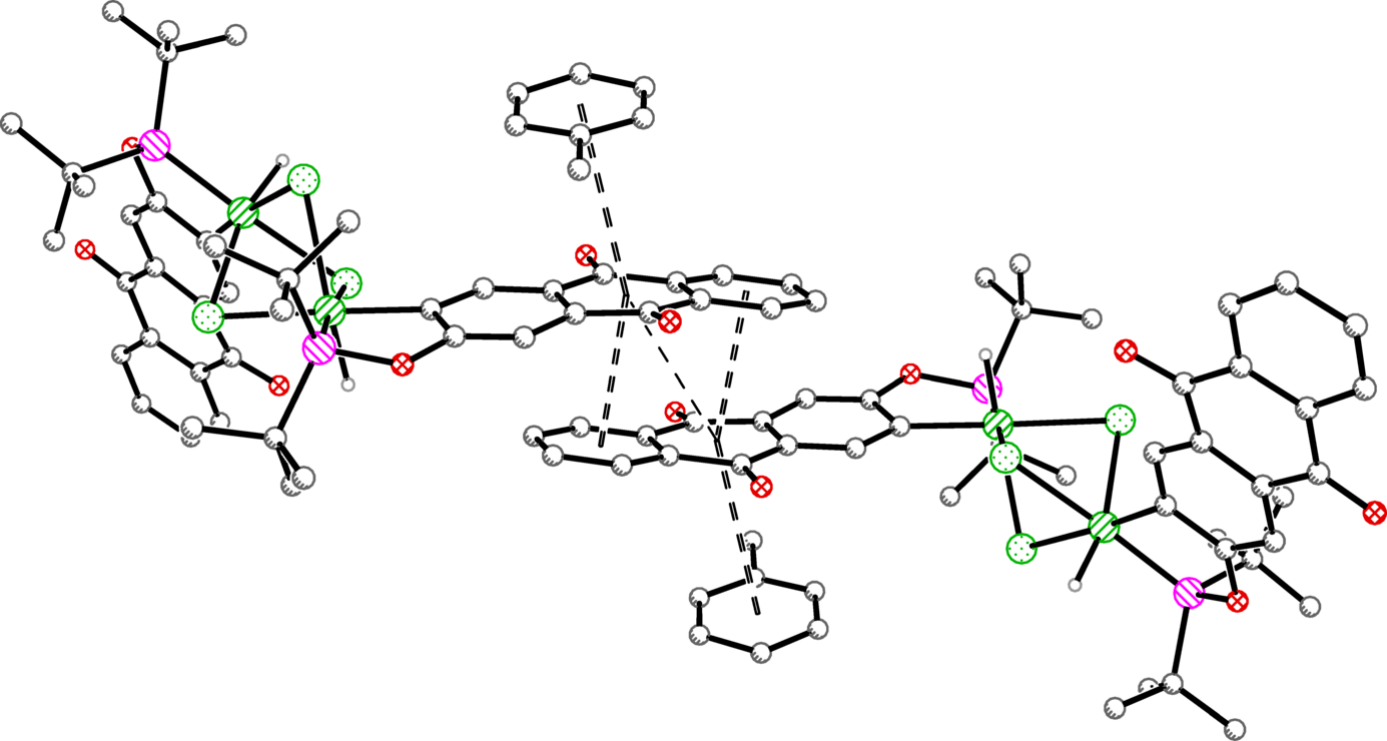
The recurring stacking of four π systems in **1**. The toluene–anthra­quinone centroid–centroid distances are 3.85 Å. The anthra­quinone–anthra­quinone centroid–centroid distances are 3.82 Å (double dashed line) and 4.63 Å (single dashed line). Symmetry equivalent generated by −*x*, 1 − *y*, 1 − *z*.

**Figure 6:**
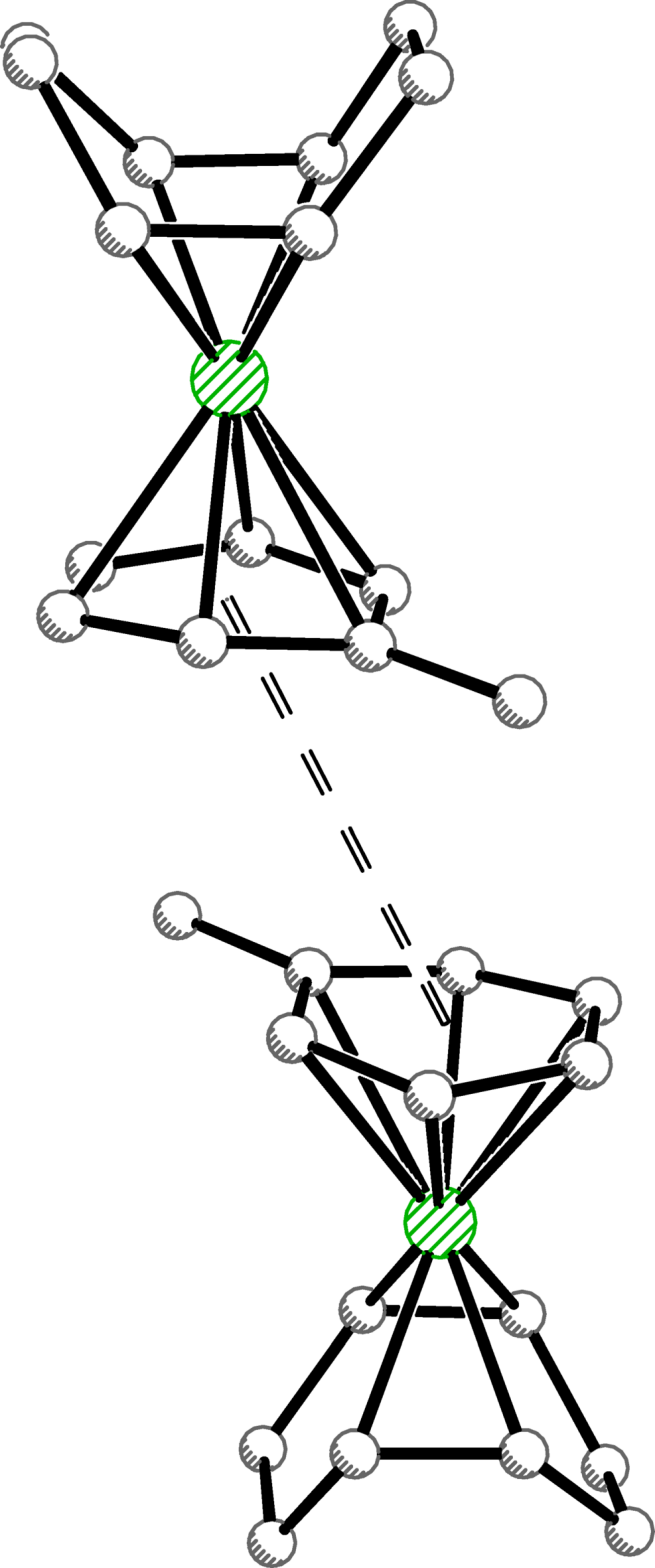
Offset parallel π-π- inter­actions of **1cat** with a centroid–centroid distance of 4.17 Å. Symmetry equivalent generated by 1 − *x*, 1 − *y*, 1 − *z*.

Mol­ecules of **1an** are inter­locked *via* offset parallel π–π inter­actions of inverted anthra­quinone groups from adjacent anions along [100] (Fig. 4 ▸). The uncoordinated toluene mol­ecules cap each anthra­quinone pairing to form a four-layer stack in the [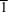
11] direction (Fig. 5 ▸). The centroid–centroid distances are 3.847 (6) Å between the toluene and anthra­quinone moieties and 3.823 (5) Å between the closest inverted anthra­quinone rings. The respective shift distances are 0.874 (12) and 1.467 (11) Å, with angles between planes of 12.8 (3) and 6.1 (3)°. [The centroid–centroid distance to the neighboring ring of the inverted anthra­quinone of 4.626 (6) Å, with its corresponding shift of 2.805 (11) Å, makes it unlikely for there to be any significant attractive force.] Inverted pairs of **1cat** fill the pockets created by the superstructure of the anions (Fig. 6 ▸), having an offset parallel orientation at a centroid–centroid distance of 4.165 (7) Å, with a shift distance of 2.003 (13) Å and angle between planes of 0° (due to symmetry). These long distances may suggest that the arrangement is a consequence of efficient packing, rather than a true attractive force. Several weak non-traditional (C—H⋯O and C—H⋯Cl) hydrogen bonds are also present (Table 3 ▸).

**Figure 7:**
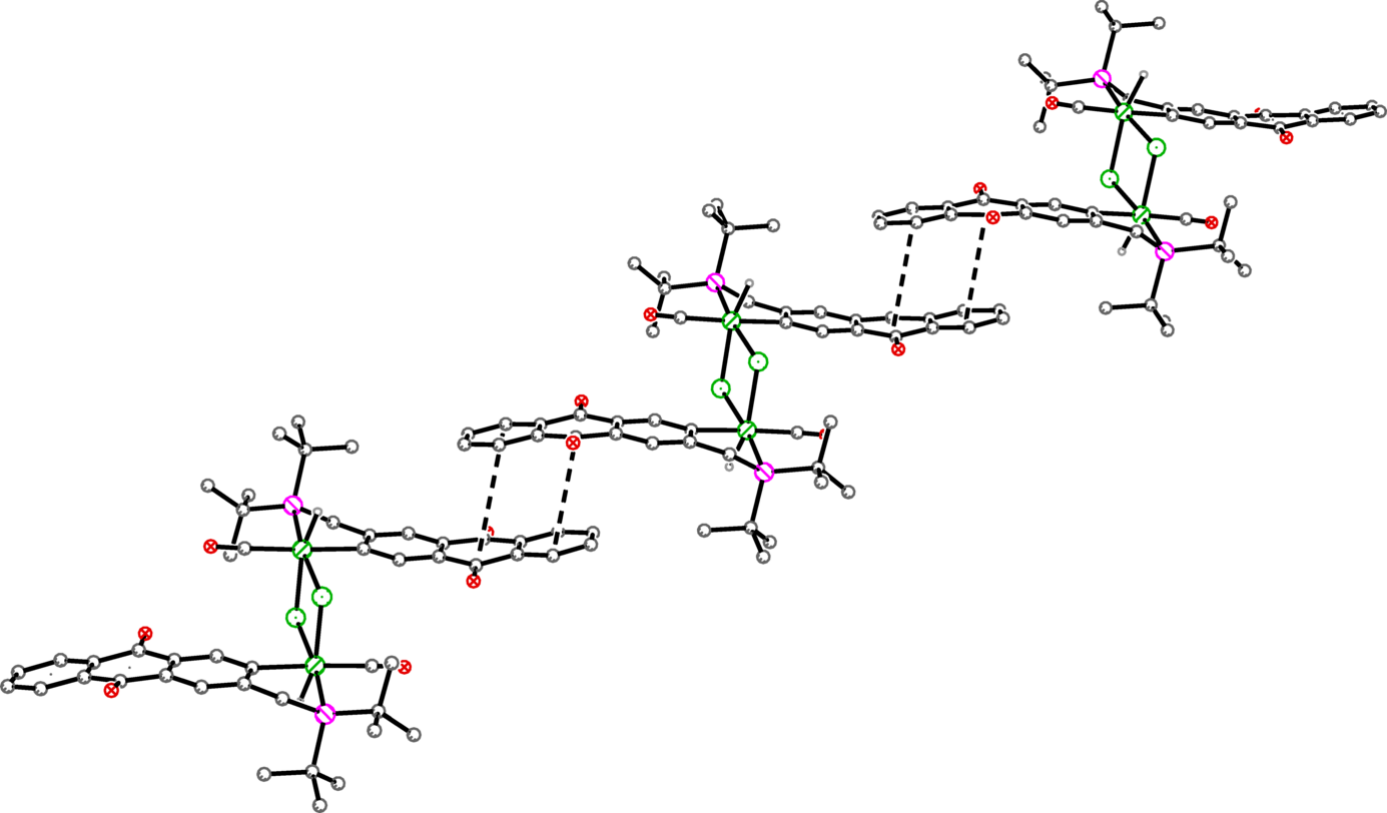
Packing plot of **2**. The mol­ecules are linked along [10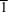] with centroid–centroid distances of 3.84 Å (double dashed line) and 3.97 Å (single dashed line).

In **2**, mol­ecules are linked in one dimension along [10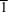] by offset parallel π–π inter­actions (Fig. 7 ▸), with centroid–centroid distances of 3.840 (2) and 3.966 (3) Å, with respective shift distances of 1.404 (6) and 1.696 (7) Å and angles between planes of 6.18 (15) and 0° (the latter exact due to symmetry). As in **1**, inter­molecular non-traditional hydrogen bonds exist (Table 4 ▸).

## 4. Database survey

To date there are ten structures containing two iridium centers bridged by three chlorido ligands: CSD refcodes GALQIT, GAMQIU (Allevi *et al.*, 1998 ▸); MOYLIV (Mura, 2000 ▸); DACCEQ, DACCIU (Zhang *et al.*, 2004 ▸); MASNEA (Yellowlees *et al.*, 2005 ▸); UCEVEE (Viciano *et al.*, 2006 ▸); YIMVIA (Dahlenburg *et al.*, 2007 ▸); KIWTUG (Dahlenburg *et al.*, 2008 ▸); PIKVAK (Tatarin *et al.*, 2023 ▸).

Structures containing an [Ir(η^6^-arene)(η^2^:η^2^-COD)]^+^ cationic unit are: CSD refcodes XIXTED (Ishii *et al.*, 2002 ▸); HUWRAS (Maekawa *et al.*, 2003 ▸); IMERAT, IMEREX, IRERIB, IMEROH, IMERUN (Muldoon & Brown, 2003 ▸); QUKLAJ, QUKLOX (Dorta *et al.*, 2004 ▸); ARACIF (Maekawa *et al.*, 2004*a* ▸); DACCEQ (Zhang *et al.*, 2004 ▸); QOMXIA (Tejel *et al.*, 2008 ▸); XOWHOI (Melcher *et al.*, 2015 ▸); KAPZOT, KAPZUZ (Drover *et al.*, 2017 ▸); BUNXEQ (Bandera *et al.*, 2020 ▸); PUFGAB (Fisher *et al.*, 2020 ▸); VUQBUH (Linden & Dorta, 2020 ▸).

Structures containing the rhodium analog of **1cat**, [Rh(η^6^-toluene)(η^2^:η^2^-COD)]^+^, are: GERKUN (Sievers *et al.*, 2022 ▸); NIDHER, NIDJOD (Sumitani *et al.*, 2023 ▸).

## 5. Synthesis and crystallization

All procedures were conducted under argon in a Vacuum Atmospheres Genesis glove box or *via* modified Schlenk techniques. All NMR spectra were collected on a JEOL JNM-ECZS 400 MHz spectrometer. All ^31^P NMR spectra were referenced to external H_3_PO_4_. ^1^H NMR spectra were referenced to residual deuterated solvent signal. All aromatic, alkane, or ether solvents were dried over sodium/benzo­phenone, distilled from the resultant purple solution prior to use, and stored over 3 Å mol­ecular sieves. CDCl_3_ and CHCl_3_ were dried/stored with 3 Å mol­ecular sieves activated by heating at 523 K under vacuum until a constant pressure of approx. 10 mTorr was reached. Methanol was dried by stirring with an excess of CaH_2_ until gas evolution through an outlet bubbler was observed to cease. It was stored over the Ca(OH)_2_ and Ca(OMe)_2_ formed, and distilled from this mixture as needed. Similarly, yellow tri­ethyl­amine obtained commercially was reacted with CaH_2_ and vacuum transferred into a Schlenk ampoule for storage as a colorless liquid. All other reagents were used as received from commercial sources without further purification.

**2-(Di-*****tert*****-butyl­phosphinito)anthra­quinone (^tBu^POAQH)**: To a 250 mL Erlenmeyer flask, 35 mg of NaH (1.46 mmol, 1.1 eq) and 75 mL of tetra­hydro­furan (THF) were added followed by 0.40 mL (2.11 mmol, 1.6 eq) of di-*tert*-butyl­chloro­phosphine and a stir bar. Then, while stirring, a solution of 300 mg (1.34 mmol) of 2-hy­droxy­anthra­quinone in 100 mL of THF was added dropwise over a period of approximately 15 minutes providing a slightly cloudy purple mixture. Slow addition of quinone and relatively dilute reaction conditions were found to be important to minimize the formation of this unknown purple byproduct. After stirring for 72 h, the reaction mixture was filtered through a Celite pad on a fine glass frit and washed with THF (2 × 5 mL). The maroon red filtrate was concentrated *in vacuo* until a brownish purple paste was obtained. The residue was stirred with toluene and refiltered to remove some reddish solids, then concentrated to dryness, and the process repeated twice more with hexane. Ultimately, a viscous green oil was obtained in 83% yield that provided NMR spectra consistent with the proposed product, ^tBu^POAQH, and of sufficient purity for further synthetic manipulations. ^31^P{^1^H} NMR (CDCl_3_): δ 158.898 (*s*). ^1^H NMR (CDCl_3_): δ 8.33–8.22 (*m*, 3H), 8.00 (*t*, *J* = 2.2 Hz, 1H), 7.81–7.72 (*m*, virtual pentet of doublets, 2H), 7.6–7.53 (*dt*, *J* = 2.5, 8.8 Hz, 1H), 1.196 (*s*, 9H), 1.165 (*s*, 9H). ^13^C NMR (CDCl_3_): δ 183.25, 182.32, 165.15, 165.06, 135.61, 134.18, 133.76, 129.98, 127.47, 127.23, 123.91, 123.79, 116.21, 116.10, 36.16, 35.90, 27.43, 27.28.

**2-(Di-*****tert*****-butyl­phosphinometh­yl)anthra­quinone (^tBu^PCA­QH)**: To a 50 mL round-bottom ampoule with a stir bar, 500 mg of 2-bromo­methyl-anthra­quinone (1.66 mmol) were added, followed by 291 mg of di-*tert*-butyl­phosphine (1.99 mmol, 1.2 eq). The vessel was then sealed, removed from the glove box, and connected to a Schlenk line. Approximately 30 mL of methanol were then added by vacuum transfer. The mixture was warmed to room temperature, and the sealed vessel then heated, with stirring, at 353 K for 72 h (note: a shorter reaction time may be possible, as all solid bromo­methyl-anthra­quinone dissolves within the first 12 h of reaction, indicative of solubilization through formation of the phospho­nium bromide salt). After heating and cooling, 1.4 mL of tri­ethyl­amine (10.0 mmol, 6 eq) were added by vacuum transfer. Upon thawing and stirring, copious formation of light solids (tri­ethyl­ammonium bromide) was observed, and the resultant mixture removed of volatiles *in vacuo*. In the glove box, the dry residue was extracted with THF and filtered through a fine frit until the NH_4_Br solids were a free-flowing powder without stickiness. The clear yellow filtrate was concentrated to apparent dryness, but still retained excess phosphine. The solids were stirred in minimal toluene, filtered, washed with hexane, and dried *in vacuo* to provide 385 mg of a lustrous yellow solid. An additional 78 mg were obtained from the filtrate stored in a freezer overnight, making the total yield 75.8%. ^31^P{^1^H} NMR (CDCl_3_): δ 39.61 (*s*). ^1^H NMR (CDCl_3_): δ 8.33–8.26 (*m*, 2H), 8.23–8.17 (*m*, 2H), 7.85–7.80 (*dvt*, 1H), 7.80–7.74 (*m*, 2H), 2.99 (*d*, *J* = 3.2, 2H), 1.17 (*s*, 9H), 1.14 (*s*, 9H). ^13^C NMR (CDCl_3_): δ 183.47, 183.11, 150.08, 149.96, 135.57, 134.12, 133.99, 133.72, 133.68, 133.41, 131.20, 128.02, 127.94, 127.52, 127.24, 127.22, 32.39, 32.17, 29.90, 29.77, 29.48, 29.23.

**[Ir(COD)(toluene)][(^tBu^POAQIrH)_2_(μ-Cl)_3_] (1)**: To a J-Young NMR tube, 15.5 mg of ^tBu^POAQH (42.07 µmol) were added followed by 14.1 mg of [Ir(COD)Cl]_2_ (21.0 µmol dimer, 1 eq of Ir). 1 mL of toluene was then added, and the sealed tube was mixed. The solution rapidly adopted a dark red–brown color. ^31^P-NMR spectroscopy in toluene revealed several products with chemical shifts at 181.5, 160.9, and 160.2 ppm. At 30 minutes after initial mixing, the singlet at 160.9 ppm was the major product, but this peak was observed to decrease with concomitant formation of small orange crystals from the solution. After 3–4 days the crystals were recovered by deca­nting the solvent and submitted for X-ray crystallographic analysis revealing the structure to be that of **1**.

**[Ir(*****κ*****-*****P***,***C*****-PCAQ)H(CO)Cl]_2_ (2)**: To a J-Young NMR tube, 12.0 mg of ^tBu^PCAQH (32.8 µmol) were added followed by 11.0 mg of [Ir(COD)Cl]_2_ (16.4 µmol dimer, 1 eq of Ir). Then, 0.75 mL of CHCl_3_ were added and the sealed tube mixed, providing an orange solution. ^31^P-NMR spectroscopy showed a species with a chemical shift of 53.0 ppm as the major product, and spectra were effectively the same after 24 h. All volatiles were then removed by vacuum, using a warm water bath once CHCl_3_ was evaporated to drive off residual excess COD. Following this, fresh CHCl_3_ (or CDCl_3_) was added by vacuum transfer and the sample then exposed to 1 atm of carbon monoxide, which caused the solution to turn pale yellow. Over a period of 1–2 days, **2** separated as yellow needles, which were isolated and submitted for X-ray diffraction studies.

## 6. Refinement

In **1**, the cation was modeled as disordered over two positions [0.894 (4):0.106 (4)]. Analogous bond lengths and angles between the two positions were restrained to be similar. Anisotropic displacement parameters for proximal atoms were restrained to be similar, and in the case of the minor component of disorder, restrained toward the expected motion relative to bond direction. The toluene solvent mol­ecule of crystallization showed signs of minor disorder. The anisotropic displacement parameters along the bonding direction between two of the atoms (C63 and C64) were restrained to be similar.

The hydrido ligands’ positions were based on peaks found in the difference-Fourier map. Once located, they were given riding models that preserved their angles relative to the other ligands, but with their Ir—H distances fixed at approximately 1.55 Å (based on an average obtained from the CSD for six-coordinate Ir complexes; Groom *et al.*, 2016 ▸). Their isotropic displacement parameters were refined relative to those of the Ir atoms: *U*_iso_(H) = 2.0*U*_eq_(Ir). Independent spectroscopic experiments confirm the presence of these ligands.

All other H atoms were placed geometrically and treated as riding atoms. Aromatic/*sp*^2^, C—H = 0.95 Å and methyl­ene, C—H = 0.99 Å, with *U*_iso_(H) = 1.2*U*_eq_(C). Methyl, C—H = 0.98 Å, with *U*_iso_(H) = 1.5*U*_eq_(C).

In **2**, reflection contributions from highly disordered solvent were fixed and added to the calculated structure factors using the SQUEEZE routine of program *PLATON* (Spek, 2015 ▸), which determined there to be 184 electrons in 492 Å^3^ treated this way per unit cell. Because the exact identity and amount of solvent were unknown, no solvent was included in the atom list or mol­ecular formula. Thus all calculated qu­anti­ties that derive from the mol­ecular formula [*e.g*., *F*(000), density, mol­ecular weight, *etc*.] are known to be inaccurate.

For **1** the maximum residual peak of 1.52 e^−^ Å^−3^ and the deepest hole of −1.72 e^−^ Å^−3^ are found 1.15 and 0.77 Å from atoms Ir1 and Ir2, respectively.

For **2** the maximum residual peak of 0.99 e^−^ Å^−3^ and the deepest hole of −1.03 e^−^ Å^−3^ are found 0.95 and 0.77 Å from atom Ir1.

Additional experimental and refinement details can be found in Table 5 ▸.

## Supplementary Material

Crystal structure: contains datablock(s) 1, 2, global. DOI: 10.1107/S2056989024008922/jy2049sup1.cif

Structure factors: contains datablock(s) 1. DOI: 10.1107/S2056989024008922/jy20491sup2.hkl

Structure factors: contains datablock(s) 2. DOI: 10.1107/S2056989024008922/jy20492sup3.hkl

CCDC references: 2383392, 2383391

Additional supporting information:  crystallographic information; 3D view; checkCIF report

## Figures and Tables

**Table 1:** Selected geometric parameters (Å, °) for **1**[Chem scheme1]

Ir1—H1*A*	1.5498	Ir3—C50	2.326 (6)
Ir1—Cl1	2.4782 (11)	Ir3—C51	2.250 (6)
Ir1—Cl2	2.4818 (11)	Ir3—C52	2.150 (6)
Ir1—Cl3	2.5819 (11)	Ir3—C53	2.139 (5)
Ir1—P1	2.1819 (11)	Ir3—C56	2.129 (6)
Ir1—C2	2.001 (5)	Ir3—C57	2.142 (7)
Ir2—H2*A*	1.5502	C45—C46	1.491 (10)
Ir2—Cl1	2.5476 (11)	C46—C47	1.393 (9)
Ir2—Cl2	2.4963 (11)	C46—C51	1.433 (9)
Ir2—Cl3	2.4780 (12)	C47—C48	1.426 (9)
Ir2—P2	2.1899 (12)	C48—C49	1.416 (9)
Ir2—C24	2.008 (5)	C49—C50	1.387 (10)
Ir3—C46	2.413 (7)	C50—C51	1.421 (9)
Ir3—C47	2.361 (7)	C52—C53	1.420 (9)
Ir3—C48	2.243 (6)	C56—C57	1.429 (10)
Ir3—C49	2.317 (6)		
			
Cl1—Ir1—H1*A*	94.5	Cl2—Ir2—Cl1	79.18 (4)
Cl1—Ir1—Cl2	80.80 (4)	Cl3—Ir2—H2*A*	91.9
Cl1—Ir1—Cl3	81.98 (4)	Cl3—Ir2—Cl1	82.68 (4)
Cl2—Ir1—H1*A*	82.5	Cl3—Ir2—Cl2	80.07 (4)
Cl2—Ir1—Cl3	78.35 (4)	P2—Ir2—H2*A*	88.6
Cl3—Ir1—H1*A*	160.9	P2—Ir2—Cl1	106.79 (4)
P1—Ir1—H1*A*	91.8	P2—Ir2—Cl2	173.06 (4)
P1—Ir1—Cl1	101.24 (4)	P2—Ir2—Cl3	103.93 (4)
P1—Ir1—Cl2	174.13 (4)	C24—Ir2—H2*A*	85.3
P1—Ir1—Cl3	107.33 (4)	C24—Ir2—Cl1	98.54 (13)
C2—Ir1—H1*A*	82.5	C24—Ir2—Cl2	93.93 (14)
C2—Ir1—Cl1	175.33 (14)	C24—Ir2—Cl3	173.57 (14)
C2—Ir1—Cl2	95.21 (13)	C24—Ir2—P2	81.82 (14)
C2—Ir1—Cl3	99.66 (14)	Ir1—Cl1—Ir2	83.38 (3)
C2—Ir1—P1	82.49 (13)	Ir1—Cl2—Ir2	84.37 (4)
Cl1—Ir2—H2*A*	164.5	Ir2—Cl3—Ir1	82.67 (4)
Cl2—Ir2—H2*A*	85.6		

**Table 2:** Selected geometric parameters (Å, °) for **2**[Chem scheme1]

Ir1—H1*A*	1.5529	Ir1—P1	2.2650 (8)
Ir1—Cl1^i^	2.5353 (8)	Ir1—C2	2.093 (3)
Ir1—Cl1	2.4537 (7)	Ir1—C24	1.932 (4)
			
Cl1—Ir1—H1*A*	88.0	C2—Ir1—P1	81.83 (10)
Cl1^i^—Ir1—H1*A*	165.1	C24—Ir1—H1*A*	94.2
Cl1—Ir1—Cl1^i^	82.39 (3)	C24—Ir1—Cl1^i^	96.98 (12)
P1—Ir1—H1*A*	88.7	C24—Ir1—Cl1	89.22 (11)
P1—Ir1—Cl1	173.11 (3)	C24—Ir1—P1	97.06 (11)
P1—Ir1—Cl1^i^	99.58 (3)	C24—Ir1—C2	177.89 (14)
C2—Ir1—H1*A*	84.0	Ir1—Cl1—Ir1^i^	97.61 (3)
C2—Ir1—Cl1	91.80 (9)		

Symmetry code: (i) 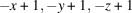.

**Table 3:** Hydrogen-bond geometry (Å, °) for **1**[Chem scheme1]

*D*—H⋯*A*	*D*—H	H⋯*A*	*D*⋯*A*	*D*—H⋯*A*
C1—H1⋯Cl2	0.95	2.72	3.348 (5)	124
C20—H20*A*⋯Cl3	0.98	2.67	3.555 (5)	151
C22—H22*C*⋯Cl3	0.98	2.74	3.613 (6)	149
C23—H23⋯Cl2	0.95	2.70	3.316 (5)	123
C38—H38*A*⋯Cl1	0.98	2.71	3.589 (6)	149
C39—H39*C*⋯Cl1	0.98	2.66	3.559 (7)	152
C7—H7⋯O5^i^	0.95	2.44	3.262 (8)	145
C16—H16*A*⋯O5^ii^	0.98	2.40	3.306 (6)	154
C45—H45*A*⋯O1^i^	0.98	2.51	3.468 (11)	167
C45—H45*C*⋯O2^iii^	0.98	2.34	3.197 (11)	145
C48—H48⋯O4^i^	1.00	2.50	3.125 (8)	120
C49—H49⋯O4^i^	1.00	2.52	3.142 (9)	120
C52—H52⋯O4^i^	1.00	2.36	3.303 (8)	157

Symmetry codes: (i) 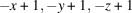; (ii) 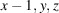; (iii) 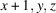.

**Table 4:** Hydrogen-bond geometry (Å, °) for **2**[Chem scheme1]

*D*—H⋯*A*	*D*—H	H⋯*A*	*D*⋯*A*	*D*—H⋯*A*
C15—H15*A*⋯O1^ii^	0.99	2.52	3.477 (5)	163
C15—H15*B*⋯O2^iii^	0.99	2.63	3.548 (4)	154
C23—H23*A*⋯Cl1^i^	0.98	2.84	3.312 (4)	111
C23—H23*B*⋯Cl1^ii^	0.98	2.86	3.757 (4)	152

Symmetry codes: (i) 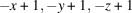; (ii) 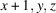; (iii) 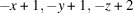.

**Table 5:** Experimental details

	**1**	**2**
Crystal data		
Chemical formula	[Ir(C_7_H_8_)(C_8_H_12_)]·[Ir_2_H_2_(C_22_H_24_O_3_P)_2_Cl_3_]·C_7_H_8_	[Ir_2_H_2_(C_23_H_26_O_2_P)_2_Cl_2_(CO)_2_]
*M*_r_	1712.17	1244.15
Crystal system, space group	Triclinic, *P* 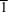	Triclinic, *P* 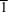
Temperature (K)	100	173
*a*, *b*, *c* (Å)	13.15698 (17), 13.58360 (18), 17.5470 (3)	8.8215 (3), 12.1331 (4), 14.4895 (3)
α, β, γ (°)	88.5722 (12), 87.0030 (12), 79.7149 (11)	81.5747 (19), 84.576 (2), 76.465 (3)
*V* (Å^3^)	3080.99 (8)	1488.57 (7)
*Z*	2	1
Radiation type	Cu *K*α	Cu *K*α
μ (mm^−1^)	14.38	10.16
Crystal size (mm)	0.10 × 0.05 × 0.01	0.19 × 0.04 × 0.03

Data collection		
Diffractometer	XtaLAB Synergy, Dualflex, HyPix	XtaLAB Synergy, Dualflex, HyPix
Absorption correction	Multi-scan (*CrysAlis PRO*; Rigaku OD, 2023 ▸)	Multi-scan (*CrysAlis PRO*; Rigaku OD, 2023 ▸)
*T*_min_, *T*_max_	0.643, 1.000	0.459, 1.000
No. of measured, independent and observed [*I* > 2σ(*I*)] reflections	51464, 12864, 11114	20510, 6137, 5626
*R*_int_	0.056	0.046
(sin θ/λ)_max_ (Å^−1^)	0.635	0.634

Refinement		
*R*[*F*^2^ > 2σ(*F*^2^)], *wR*(*F*^2^), *S*	0.035, 0.092, 1.04	0.027, 0.067, 1.06
No. of reflections	12864	6137
No. of parameters	881	277
No. of restraints	395	0
H-atom treatment	H-atom parameters constrained	H-atom parameters constrained
Δρ_max_, Δρ_min_ (e Å^−3^)	1.52, −1.72	0.99, −1.03

Computer programs: *CrysAlis PRO* (Rigaku OD, 2023 ▸), *SHELXT2018/2* (Sheldrick, 2015*a* ▸), *SHELXL2019/3* (Sheldrick, 2015*b* ▸) and *OLEX2* (Dolomanov *et al.*, 2009 ▸).

## supplementary crystallographic information

### (η2:η2-Cycloocta-1,5-diene)(η6-toluene)iridium(I) tri-µ-chlorido-bis({3-[(di-tert-butylphosphanyl)oxy]-9,10-dioxoanthracen-2-yl}hydridoiridium(III)) toluene monosolvate, (1) Crystal data

**Table:**

[Ir(C_7_H_8_)(C_8_H_12_)]·[Ir_2_H_2_(C_22_H_24_O_3_P)_2_Cl_3_]·C_7_H_8_	*Z* = 2
*M**_r_* = 1712.17	*F*(000) = 1668
Triclinic, *P*1	*D*_x_ = 1.846 Mg m^−^^3^
*a* = 13.15698 (17) Å	Cu *K*α radiation, λ = 1.54184 Å
*b* = 13.58360 (18) Å	Cell parameters from 23439 reflections
*c* = 17.5470 (3) Å	θ = 2.6–77.6°
α = 88.5722 (12)°	µ = 14.38 mm^−^^1^
β = 87.0030 (12)°	*T* = 100 K
γ = 79.7149 (11)°	Plate, orange
*V* = 3080.99 (8) Å^3^	0.10 × 0.05 × 0.01 mm

### (η2:η2-Cycloocta-1,5-diene)(η6-toluene)iridium(I) tri-µ-chlorido-bis({3-[(di-tert-butylphosphanyl)oxy]-9,10-dioxoanthracen-2-yl}hydridoiridium(III)) toluene monosolvate, (1) Data collection

**Table:**

XtaLAB Synergy, Dualflex, HyPix diffractometer	12864 independent reflections
Radiation source: micro-focus sealed X-ray tube, PhotonJet (Cu) X-ray Source	11114 reflections with *I* > 2σ(*I*)
Mirror monochromator	*R*_int_ = 0.056
Detector resolution: 10.0000 pixels mm^-1^	θ_max_ = 78.2°, θ_min_ = 2.5°
ω scans	*h* = −16→14
Absorption correction: multi-scan (CrysAlisPro; Rigaku OD, 2023)	*k* = −16→17
*T*_min_ = 0.643, *T*_max_ = 1.000	*l* = −22→21
51464 measured reflections	

### (η2:η2-Cycloocta-1,5-diene)(η6-toluene)iridium(I) tri-µ-chlorido-bis({3-[(di-tert-butylphosphanyl)oxy]-9,10-dioxoanthracen-2-yl}hydridoiridium(III)) toluene monosolvate, (1) Refinement

**Table:**

Refinement on *F*^2^	Primary atom site location: dual
Least-squares matrix: full	Secondary atom site location: difference Fourier map
*R*[*F*^2^ > 2σ(*F*^2^)] = 0.035	Hydrogen site location: mixed
*wR*(*F*^2^) = 0.092	H-atom parameters constrained
*S* = 1.04	*w* = 1/[σ^2^(*F*_o_^2^) + (0.0514*P*)^2^ + 3.1792*P*] where *P* = (*F*_o_^2^ + 2*F*_c_^2^)/3
12864 reflections	(Δ/σ)_max_ = 0.002
881 parameters	Δρ_max_ = 1.52 e Å^−^^3^
395 restraints	Δρ_min_ = −1.72 e Å^−^^3^

### (η2:η2-Cycloocta-1,5-diene)(η6-toluene)iridium(I) tri-µ-chlorido-bis({3-[(di-tert-butylphosphanyl)oxy]-9,10-dioxoanthracen-2-yl}hydridoiridium(III)) toluene monosolvate, (1) Special details

**Table:**

Geometry. All esds (except the esd in the dihedral angle between two l.s. planes) are estimated using the full covariance matrix. The cell esds are taken into account individually in the estimation of esds in distances, angles and torsion angles; correlations between esds in cell parameters are only used when they are defined by crystal symmetry. An approximate (isotropic) treatment of cell esds is used for estimating esds involving l.s. planes.
Refinement. The [Ir(toluene)(COD)]^+^ cation is modeled as disordered over two positions (0.894 (4):0.106 (4)). Analogous bond lengths and angles between the two positions of the disordered cation were restrained to be similar. Anisotropic displacement parameters for proximal atoms were restrained to be similar, and in the case of the minor component of disorder, restrained toward the expected motion relative to bond direction.The hydrido ligands' positions were based on a peaks found in the difference Fourier map. Once located, they were given riding models that preserved their angles relative to the other ligands, but with their Ir–H distances fixed at approximately 1.55 Å (based on an average obtained from the Cambridge Structural Database for six-coordinate Ir complexes; Groom *et al.*, 2016). Independent spectroscopic experiments confirm the presence of these ligands.The toluene solvent molecule of crystallization showed signs of minor disorder. The anisotropic displacement parameters along the bonding direction between two of the atoms (C63 and C64) were restrained to be similar.

### (η2:η2-Cycloocta-1,5-diene)(η6-toluene)iridium(I) tri-µ-chlorido-bis({3-[(di-tert-butylphosphanyl)oxy]-9,10-dioxoanthracen-2-yl}hydridoiridium(III)) toluene monosolvate, (1) Fractional atomic coordinates and isotropic or equivalent isotropic displacement parameters (Å^2^)

**Table:**

	*x*	*y*	*z*	*U*_iso_*/*U*_eq_	Occ. (<1)
Ir1	0.25577 (2)	0.75554 (2)	0.22505 (2)	0.02091 (6)	
H1A	0.200526	0.820299	0.291646	0.042*	
Ir2	0.51415 (2)	0.71861 (2)	0.21257 (2)	0.02152 (6)	
H2A	0.575351	0.618205	0.242631	0.043*	
Cl1	0.37608 (8)	0.87185 (8)	0.18895 (7)	0.0265 (2)	
Cl2	0.38742 (8)	0.70260 (8)	0.32111 (7)	0.0242 (2)	
Cl3	0.39277 (8)	0.63198 (9)	0.14812 (7)	0.0269 (2)	
P1	0.12927 (8)	0.80830 (9)	0.15027 (7)	0.0218 (2)	
P2	0.63977 (9)	0.72368 (9)	0.12642 (7)	0.0238 (2)	
O1	0.2189 (3)	0.4434 (4)	0.4379 (3)	0.0477 (11)	
O2	−0.1358 (3)	0.4740 (3)	0.2948 (3)	0.0399 (9)	
O3	0.0327 (2)	0.7541 (3)	0.1832 (2)	0.0254 (7)	
O4	0.5391 (3)	0.8854 (3)	0.4990 (2)	0.0318 (8)	
O5	0.9140 (3)	0.9115 (3)	0.3714 (2)	0.0345 (8)	
O6	0.7382 (2)	0.7444 (3)	0.1751 (2)	0.0259 (7)	
C1	0.1828 (4)	0.5875 (4)	0.3189 (3)	0.0290 (11)	
H1	0.248452	0.576864	0.340871	0.035*	
C2	0.1620 (3)	0.6622 (4)	0.2630 (3)	0.0219 (9)	
C3	0.0611 (3)	0.6751 (4)	0.2347 (3)	0.0232 (9)	
C4	−0.0100 (4)	0.6159 (4)	0.2553 (3)	0.0269 (10)	
H4	−0.075102	0.625960	0.232566	0.032*	
C5	−0.1155 (4)	0.3549 (5)	0.4283 (4)	0.0384 (13)	
H5	−0.178019	0.358876	0.402726	0.046*	
C6	−0.0994 (5)	0.2969 (5)	0.4940 (4)	0.0432 (15)	
H6	−0.151799	0.262042	0.513649	0.052*	
C7	−0.0083 (5)	0.2890 (5)	0.5315 (4)	0.0432 (15)	
H7	0.001919	0.248665	0.576399	0.052*	
C8	0.0686 (5)	0.3409 (5)	0.5029 (4)	0.0407 (14)	
H8	0.131092	0.336087	0.528613	0.049*	
C9	0.1358 (4)	0.4560 (4)	0.4080 (3)	0.0338 (12)	
C10	−0.0594 (4)	0.4743 (4)	0.3314 (3)	0.0309 (11)	
C11	0.1113 (4)	0.5278 (4)	0.3439 (3)	0.0287 (10)	
C12	0.0148 (4)	0.5402 (4)	0.3106 (3)	0.0269 (10)	
C13	−0.0392 (4)	0.4075 (4)	0.3996 (4)	0.0352 (12)	
C14	0.0540 (4)	0.3994 (4)	0.4368 (3)	0.0333 (12)	
C15	0.0663 (4)	0.9437 (4)	0.1536 (3)	0.0276 (10)	
C16	0.0692 (4)	0.9819 (4)	0.2344 (3)	0.0343 (12)	
H16A	0.035645	0.940129	0.270458	0.051*	
H16B	0.032507	1.051296	0.237085	0.051*	
H16C	0.141194	0.978883	0.247395	0.051*	
C17	−0.0479 (4)	0.9603 (4)	0.1335 (3)	0.0328 (12)	
H17A	−0.052731	0.940503	0.080626	0.049*	
H17B	−0.078391	1.031206	0.139047	0.049*	
H17C	−0.085441	0.919815	0.167879	0.049*	
C18	0.1285 (4)	1.0042 (4)	0.0998 (3)	0.0319 (11)	
H18A	0.200820	0.992344	0.113981	0.048*	
H18B	0.099540	1.075578	0.104014	0.048*	
H18C	0.124979	0.983176	0.047134	0.048*	
C19	0.1460 (4)	0.7634 (4)	0.0488 (3)	0.0286 (10)	
C20	0.1557 (4)	0.6497 (4)	0.0560 (3)	0.0322 (11)	
H20A	0.211691	0.623056	0.089562	0.048*	
H20B	0.170972	0.620025	0.005442	0.048*	
H20C	0.090494	0.633072	0.077632	0.048*	
C21	0.0535 (4)	0.8025 (5)	0.0007 (3)	0.0351 (12)	
H21A	−0.010146	0.790646	0.028197	0.053*	
H21B	0.061724	0.767438	−0.047946	0.053*	
H21C	0.049438	0.874408	−0.008899	0.053*	
C22	0.2444 (4)	0.7907 (5)	0.0101 (3)	0.0354 (12)	
H22A	0.236125	0.863501	0.003844	0.053*	
H22B	0.256729	0.759482	−0.040132	0.053*	
H22C	0.303263	0.766404	0.041629	0.053*	
C23	0.5809 (3)	0.8226 (4)	0.3475 (3)	0.0250 (10)	
H23	0.513172	0.823092	0.369294	0.030*	
C24	0.6063 (3)	0.7829 (4)	0.2759 (3)	0.0240 (9)	
C25	0.7091 (3)	0.7841 (4)	0.2466 (3)	0.0235 (9)	
C26	0.7803 (4)	0.8233 (4)	0.2854 (3)	0.0280 (10)	
H26	0.847888	0.823162	0.263414	0.034*	
C27	0.8549 (4)	1.0097 (4)	0.5097 (3)	0.0327 (12)	
H27	0.920606	1.015387	0.486771	0.039*	
C28	0.8241 (4)	1.0534 (5)	0.5787 (4)	0.0359 (12)	
H28	0.868444	1.089525	0.603055	0.043*	
C29	0.7280 (4)	1.0450 (4)	0.6135 (3)	0.0344 (12)	
H29	0.706971	1.074717	0.661520	0.041*	
C30	0.6634 (4)	0.9929 (4)	0.5769 (3)	0.0321 (11)	
H30	0.598029	0.987061	0.600445	0.039*	
C31	0.6206 (4)	0.8984 (4)	0.4674 (3)	0.0258 (10)	
C32	0.8253 (3)	0.9098 (4)	0.3981 (3)	0.0253 (10)	
C33	0.6517 (3)	0.8620 (4)	0.3890 (3)	0.0241 (9)	
C34	0.7521 (3)	0.8630 (4)	0.3574 (3)	0.0256 (10)	
C35	0.7904 (4)	0.9568 (4)	0.4730 (3)	0.0257 (10)	
C36	0.6928 (4)	0.9491 (4)	0.5066 (3)	0.0252 (10)	
C37	0.6257 (4)	0.8327 (4)	0.0585 (3)	0.0316 (11)	
C38	0.6159 (4)	0.9265 (4)	0.1071 (4)	0.0368 (13)	
H38A	0.558959	0.927409	0.145550	0.055*	
H38B	0.601814	0.986308	0.074242	0.055*	
H38C	0.680550	0.925745	0.132544	0.055*	
C39	0.5265 (4)	0.8376 (5)	0.0140 (4)	0.0386 (13)	
H39A	0.532472	0.777442	−0.016811	0.058*	
H39B	0.517589	0.896987	−0.019527	0.058*	
H39C	0.466597	0.841449	0.050102	0.058*	
C40	0.7175 (4)	0.8323 (5)	0.0005 (4)	0.0411 (14)	
H40A	0.781491	0.826642	0.027765	0.062*	
H40B	0.707022	0.894728	−0.029583	0.062*	
H40C	0.722507	0.775370	−0.033513	0.062*	
C41	0.7012 (4)	0.6053 (4)	0.0773 (3)	0.0281 (10)	
C42	0.6353 (4)	0.5881 (5)	0.0106 (3)	0.0368 (12)	
H42A	0.563300	0.590912	0.029217	0.055*	
H42B	0.661855	0.522233	−0.011267	0.055*	
H42C	0.638704	0.640103	−0.028703	0.055*	
C43	0.8139 (4)	0.6072 (4)	0.0506 (3)	0.0337 (12)	
H43A	0.815390	0.655780	0.008477	0.051*	
H43B	0.846810	0.540553	0.033082	0.051*	
H43C	0.851367	0.626359	0.093025	0.051*	
C44	0.7018 (4)	0.5187 (4)	0.1344 (3)	0.0337 (11)	
H44A	0.739810	0.530603	0.178810	0.051*	
H44B	0.735440	0.456245	0.110208	0.051*	
H44C	0.630508	0.513737	0.150711	0.051*	
Ir3	0.59560 (5)	0.30166 (4)	0.35208 (4)	0.02601 (17)	0.893 (4)
C45	0.6547 (7)	0.5413 (6)	0.3954 (6)	0.040 (2)	0.893 (4)
H45A	0.700041	0.537809	0.438291	0.061*	0.893 (4)
H45B	0.611717	0.607990	0.392760	0.061*	0.893 (4)
H45C	0.696851	0.528569	0.347738	0.061*	0.893 (4)
C46	0.5870 (5)	0.4645 (5)	0.4065 (4)	0.0337 (14)	0.893 (4)
C47	0.5991 (5)	0.3923 (5)	0.4647 (4)	0.0349 (15)	0.893 (4)
H47	0.663832	0.381605	0.493543	0.042*	0.893 (4)
C48	0.5321 (5)	0.3206 (5)	0.4730 (4)	0.0317 (13)	0.893 (4)
H48	0.546683	0.263986	0.510642	0.038*	0.893 (4)
C49	0.4443 (5)	0.3302 (6)	0.4282 (4)	0.0389 (16)	0.893 (4)
H49	0.399030	0.278260	0.432450	0.047*	0.893 (4)
C50	0.4326 (5)	0.4003 (5)	0.3690 (4)	0.0370 (16)	0.893 (4)
H50	0.379430	0.397487	0.330781	0.044*	0.893 (4)
C51	0.5093 (5)	0.4604 (5)	0.3530 (4)	0.0331 (14)	0.893 (4)
H51	0.507361	0.502160	0.305146	0.040*	0.893 (4)
C52	0.6116 (5)	0.1415 (4)	0.3482 (4)	0.0313 (13)	0.893 (4)
H52	0.573160	0.112574	0.391317	0.038*	0.893 (4)
C53	0.7058 (5)	0.1678 (4)	0.3695 (4)	0.0336 (13)	0.893 (4)
H53	0.722146	0.152516	0.423969	0.040*	0.893 (4)
C54	0.8000 (5)	0.1546 (7)	0.3136 (6)	0.0523 (19)	0.893 (4)
H54A	0.863328	0.145756	0.342924	0.063*	0.893 (4)
H54B	0.801965	0.092786	0.284381	0.063*	0.893 (4)
C55	0.8008 (6)	0.2410 (7)	0.2584 (5)	0.0466 (19)	0.893 (4)
H55A	0.821145	0.214246	0.206760	0.056*	0.893 (4)
H55B	0.853834	0.279339	0.272934	0.056*	0.893 (4)
C56	0.6976 (5)	0.3117 (5)	0.2550 (4)	0.0361 (15)	0.893 (4)
H56	0.703951	0.381872	0.239902	0.043*	0.893 (4)
C57	0.6055 (6)	0.2822 (5)	0.2310 (4)	0.0377 (15)	0.893 (4)
H57	0.559288	0.334565	0.201636	0.045*	0.893 (4)
C58	0.6050 (9)	0.1761 (8)	0.2083 (5)	0.058 (3)	0.893 (4)
H58A	0.548104	0.176589	0.173437	0.070*	0.893 (4)
H58B	0.670677	0.151113	0.179211	0.070*	0.893 (4)
C59	0.5926 (8)	0.1038 (5)	0.2724 (5)	0.050 (2)	0.893 (4)
H59A	0.521543	0.088977	0.273540	0.060*	0.893 (4)
H59B	0.641296	0.040419	0.262702	0.060*	0.893 (4)
C60	0.0170 (5)	0.3969 (6)	0.1369 (5)	0.0545 (17)	
H60A	0.011193	0.469799	0.138247	0.082*	
H60B	−0.045156	0.377596	0.161653	0.082*	
H60C	0.024055	0.375211	0.083735	0.082*	
C61	0.1093 (5)	0.3487 (5)	0.1778 (4)	0.0501 (16)	
C62	0.2009 (6)	0.3886 (7)	0.1694 (4)	0.0536 (17)	
H62	0.203041	0.447109	0.138911	0.064*	
C63	0.2866 (6)	0.3430 (8)	0.2052 (5)	0.070 (2)	
H63	0.348874	0.368942	0.198027	0.083*	
C64	0.2849 (8)	0.2590 (8)	0.2521 (8)	0.092 (4)	
H64	0.346050	0.226756	0.275138	0.111*	
C65	0.1908 (9)	0.2218 (8)	0.2653 (9)	0.103 (4)	
H65	0.186654	0.166647	0.298910	0.123*	
C66	0.1065 (7)	0.2689 (6)	0.2275 (7)	0.077 (3)	
H66	0.043053	0.245212	0.235897	0.092*	
Ir3A	0.6214 (7)	0.2903 (5)	0.3322 (5)	0.069 (2)	0.107 (4)
C52A	0.637 (3)	0.1291 (16)	0.338 (2)	0.0316 (16)	0.107 (4)
H52A	0.597261	0.105478	0.382680	0.038*	0.107 (4)
C56A	0.722 (3)	0.292 (3)	0.2370 (19)	0.039 (5)	0.107 (4)
H56A	0.729985	0.360929	0.218427	0.047*	0.107 (4)
C45A	0.666 (6)	0.537 (4)	0.373 (4)	0.038 (13)	0.107 (4)
H45D	0.628011	0.598276	0.396124	0.056*	0.107 (4)
H45E	0.679895	0.549947	0.318044	0.056*	0.107 (4)
H45F	0.732190	0.516921	0.397171	0.056*	0.107 (4)
C54A	0.826 (3)	0.142 (5)	0.304 (4)	0.052 (2)	0.107 (4)
H54C	0.886908	0.144399	0.334067	0.063*	0.107 (4)
H54D	0.834673	0.073773	0.282491	0.063*	0.107 (4)
C46A	0.605 (3)	0.456 (2)	0.382 (2)	0.034 (6)	0.107 (4)
C58A	0.625 (8)	0.153 (4)	0.197 (3)	0.057 (7)	0.107 (4)
H58C	0.567471	0.152434	0.163411	0.068*	0.107 (4)
H58D	0.690078	0.124756	0.168237	0.068*	0.107 (4)
C47A	0.617 (3)	0.387 (3)	0.4436 (17)	0.035 (6)	0.107 (4)
H47A	0.679890	0.380649	0.473973	0.042*	0.107 (4)
C53A	0.729 (3)	0.158 (2)	0.358 (2)	0.0339 (16)	0.107 (4)
H53A	0.743516	0.148377	0.412891	0.041*	0.107 (4)
C48A	0.552 (3)	0.316 (3)	0.4535 (16)	0.035 (5)	0.107 (4)
H48A	0.566997	0.261589	0.492958	0.042*	0.107 (4)
C55A	0.823 (3)	0.217 (5)	0.239 (3)	0.053 (9)	0.107 (4)
H55C	0.834107	0.180631	0.190662	0.063*	0.107 (4)
H55D	0.880598	0.253857	0.243706	0.063*	0.107 (4)
C49A	0.464 (2)	0.320 (3)	0.408 (2)	0.035 (5)	0.107 (4)
H49A	0.420095	0.266978	0.414151	0.042*	0.107 (4)
C57A	0.629 (3)	0.261 (3)	0.2140 (14)	0.041 (5)	0.107 (4)
H57A	0.583304	0.311168	0.182891	0.049*	0.107 (4)
C50A	0.453 (2)	0.387 (3)	0.348 (2)	0.032 (5)	0.107 (4)
H50A	0.401419	0.379886	0.309595	0.038*	0.107 (4)
C59A	0.612 (7)	0.087 (3)	0.265 (3)	0.050 (2)	0.107 (4)
H59C	0.539893	0.075688	0.269041	0.060*	0.107 (4)
H59D	0.657796	0.021540	0.257114	0.060*	0.107 (4)
C51A	0.530 (3)	0.4465 (19)	0.327 (2)	0.035 (5)	0.107 (4)
H51A	0.526317	0.486248	0.278557	0.042*	0.107 (4)

### (η2:η2-Cycloocta-1,5-diene)(η6-toluene)iridium(I) tri-µ-chlorido-bis({3-[(di-tert-butylphosphanyl)oxy]-9,10-dioxoanthracen-2-yl}hydridoiridium(III)) toluene monosolvate, (1) Atomic displacement parameters (Å^2^)

**Table:**

	*U*^11^	*U*^22^	*U*^33^	*U*^12^	*U*^13^	*U*^23^
Ir1	0.01439 (9)	0.02208 (10)	0.02629 (11)	−0.00396 (7)	−0.00011 (7)	0.00233 (7)
Ir2	0.01490 (9)	0.02194 (10)	0.02730 (11)	−0.00311 (7)	0.00044 (7)	0.00366 (8)
Cl1	0.0178 (5)	0.0255 (5)	0.0361 (6)	−0.0045 (4)	0.0007 (4)	0.0045 (4)
Cl2	0.0165 (4)	0.0266 (5)	0.0297 (6)	−0.0050 (4)	−0.0009 (4)	0.0031 (4)
Cl3	0.0198 (5)	0.0274 (5)	0.0328 (6)	−0.0032 (4)	−0.0001 (4)	−0.0010 (4)
P1	0.0158 (5)	0.0248 (5)	0.0247 (6)	−0.0039 (4)	−0.0001 (4)	0.0046 (4)
P2	0.0174 (5)	0.0250 (6)	0.0278 (6)	−0.0026 (4)	0.0028 (4)	0.0051 (5)
O1	0.030 (2)	0.061 (3)	0.054 (3)	−0.0140 (19)	−0.0111 (18)	0.026 (2)
O2	0.0314 (19)	0.045 (2)	0.048 (2)	−0.0200 (17)	−0.0083 (17)	0.0150 (19)
O3	0.0159 (14)	0.0263 (17)	0.0347 (19)	−0.0059 (12)	−0.0037 (13)	0.0085 (14)
O4	0.0244 (17)	0.040 (2)	0.034 (2)	−0.0137 (15)	0.0033 (14)	−0.0011 (16)
O5	0.0185 (16)	0.048 (2)	0.038 (2)	−0.0112 (15)	−0.0021 (14)	0.0040 (18)
O6	0.0166 (14)	0.0289 (17)	0.0303 (18)	−0.0004 (13)	0.0030 (13)	0.0016 (14)
C1	0.019 (2)	0.033 (3)	0.037 (3)	−0.0092 (19)	−0.0048 (19)	0.007 (2)
C2	0.0170 (19)	0.026 (2)	0.023 (2)	−0.0043 (17)	−0.0005 (16)	−0.0011 (18)
C3	0.020 (2)	0.025 (2)	0.024 (2)	−0.0030 (17)	0.0017 (17)	0.0030 (18)
C4	0.022 (2)	0.030 (2)	0.030 (3)	−0.0043 (19)	−0.0065 (18)	0.002 (2)
C5	0.033 (3)	0.039 (3)	0.045 (3)	−0.011 (2)	−0.001 (2)	0.011 (3)
C6	0.035 (3)	0.036 (3)	0.057 (4)	−0.007 (2)	0.005 (3)	0.020 (3)
C7	0.038 (3)	0.040 (3)	0.048 (4)	−0.005 (2)	0.003 (3)	0.020 (3)
C8	0.032 (3)	0.037 (3)	0.051 (4)	−0.004 (2)	0.002 (2)	0.015 (3)
C9	0.026 (2)	0.038 (3)	0.037 (3)	−0.006 (2)	−0.005 (2)	0.012 (2)
C10	0.021 (2)	0.035 (3)	0.038 (3)	−0.011 (2)	−0.003 (2)	0.010 (2)
C11	0.020 (2)	0.034 (3)	0.032 (3)	−0.0069 (19)	−0.0017 (19)	0.007 (2)
C12	0.020 (2)	0.030 (2)	0.032 (3)	−0.0066 (18)	−0.0026 (18)	0.005 (2)
C13	0.032 (3)	0.031 (3)	0.044 (3)	−0.012 (2)	0.000 (2)	0.011 (2)
C14	0.026 (2)	0.039 (3)	0.034 (3)	−0.004 (2)	0.000 (2)	0.013 (2)
C15	0.023 (2)	0.026 (2)	0.033 (3)	−0.0049 (18)	0.0003 (19)	0.006 (2)
C16	0.032 (3)	0.031 (3)	0.038 (3)	0.000 (2)	0.002 (2)	0.006 (2)
C17	0.020 (2)	0.032 (3)	0.043 (3)	0.0016 (19)	0.002 (2)	0.012 (2)
C18	0.025 (2)	0.027 (2)	0.045 (3)	−0.0060 (19)	−0.001 (2)	0.009 (2)
C19	0.027 (2)	0.032 (3)	0.026 (2)	−0.0041 (19)	0.0031 (19)	0.000 (2)
C20	0.030 (2)	0.030 (3)	0.036 (3)	−0.004 (2)	−0.007 (2)	−0.003 (2)
C21	0.032 (3)	0.041 (3)	0.032 (3)	−0.007 (2)	−0.007 (2)	−0.002 (2)
C22	0.033 (3)	0.044 (3)	0.029 (3)	−0.006 (2)	0.002 (2)	0.006 (2)
C23	0.016 (2)	0.023 (2)	0.035 (3)	−0.0021 (17)	0.0035 (18)	0.0057 (19)
C24	0.016 (2)	0.025 (2)	0.029 (2)	−0.0017 (17)	0.0019 (17)	0.0041 (19)
C25	0.016 (2)	0.024 (2)	0.031 (2)	−0.0036 (17)	0.0003 (17)	0.0052 (19)
C26	0.018 (2)	0.030 (2)	0.035 (3)	−0.0036 (18)	0.0027 (18)	0.008 (2)
C27	0.022 (2)	0.034 (3)	0.044 (3)	−0.008 (2)	−0.011 (2)	0.012 (2)
C28	0.028 (3)	0.040 (3)	0.042 (3)	−0.012 (2)	−0.012 (2)	0.001 (2)
C29	0.025 (2)	0.039 (3)	0.039 (3)	−0.003 (2)	−0.004 (2)	−0.005 (2)
C30	0.021 (2)	0.036 (3)	0.040 (3)	−0.006 (2)	−0.002 (2)	−0.001 (2)
C31	0.019 (2)	0.026 (2)	0.031 (3)	−0.0036 (18)	−0.0001 (18)	0.005 (2)
C32	0.015 (2)	0.028 (2)	0.033 (3)	−0.0055 (17)	−0.0037 (17)	0.011 (2)
C33	0.0147 (19)	0.022 (2)	0.036 (3)	−0.0048 (16)	0.0007 (18)	0.0024 (19)
C34	0.017 (2)	0.026 (2)	0.033 (3)	−0.0026 (17)	−0.0002 (18)	0.008 (2)
C35	0.021 (2)	0.027 (2)	0.031 (3)	−0.0062 (18)	−0.0071 (18)	0.007 (2)
C36	0.020 (2)	0.021 (2)	0.033 (3)	−0.0011 (17)	−0.0022 (18)	0.0070 (19)
C37	0.024 (2)	0.032 (3)	0.038 (3)	−0.004 (2)	0.001 (2)	0.012 (2)
C38	0.026 (2)	0.028 (3)	0.055 (4)	−0.006 (2)	0.000 (2)	0.013 (2)
C39	0.031 (3)	0.042 (3)	0.041 (3)	−0.004 (2)	0.000 (2)	0.015 (3)
C40	0.029 (3)	0.039 (3)	0.051 (4)	0.000 (2)	0.008 (2)	0.015 (3)
C41	0.022 (2)	0.028 (2)	0.033 (3)	−0.0023 (19)	0.0013 (19)	0.001 (2)
C42	0.032 (3)	0.042 (3)	0.035 (3)	−0.005 (2)	0.001 (2)	0.001 (2)
C43	0.022 (2)	0.040 (3)	0.037 (3)	0.000 (2)	0.009 (2)	−0.003 (2)
C44	0.033 (3)	0.029 (3)	0.036 (3)	−0.001 (2)	0.004 (2)	0.001 (2)
Ir3	0.0214 (2)	0.02444 (16)	0.0315 (3)	−0.00303 (13)	−0.00204 (15)	0.00769 (15)
C45	0.045 (4)	0.036 (4)	0.038 (6)	−0.002 (3)	−0.001 (4)	−0.001 (3)
C46	0.032 (3)	0.034 (3)	0.032 (4)	0.000 (2)	0.004 (3)	0.000 (3)
C47	0.034 (3)	0.039 (3)	0.029 (3)	−0.002 (3)	0.001 (3)	0.005 (3)
C48	0.036 (3)	0.032 (3)	0.025 (3)	−0.003 (2)	0.010 (2)	0.005 (2)
C49	0.023 (3)	0.045 (4)	0.046 (4)	−0.003 (3)	0.010 (3)	−0.001 (3)
C50	0.024 (3)	0.033 (3)	0.049 (4)	0.008 (2)	0.001 (3)	−0.004 (3)
C51	0.027 (3)	0.024 (3)	0.043 (4)	0.008 (2)	0.002 (3)	0.002 (3)
C52	0.031 (3)	0.018 (2)	0.043 (3)	−0.002 (2)	0.007 (2)	0.003 (2)
C53	0.027 (3)	0.021 (2)	0.049 (4)	0.006 (2)	−0.003 (2)	0.011 (2)
C54	0.027 (4)	0.042 (4)	0.084 (5)	−0.004 (3)	0.009 (3)	0.014 (4)
C55	0.037 (4)	0.059 (5)	0.042 (4)	−0.007 (3)	0.012 (3)	−0.001 (4)
C56	0.033 (3)	0.032 (3)	0.041 (4)	−0.006 (3)	0.013 (3)	0.012 (3)
C57	0.051 (4)	0.040 (4)	0.021 (3)	−0.002 (3)	−0.002 (3)	0.000 (3)
C58	0.078 (7)	0.068 (6)	0.041 (4)	−0.046 (6)	0.001 (4)	−0.008 (4)
C59	0.064 (6)	0.031 (4)	0.053 (4)	−0.001 (4)	−0.011 (4)	−0.007 (3)
C60	0.045 (4)	0.055 (4)	0.062 (5)	−0.004 (3)	0.000 (3)	−0.016 (3)
C61	0.046 (3)	0.047 (4)	0.057 (4)	−0.008 (3)	0.004 (3)	−0.020 (3)
C62	0.052 (4)	0.070 (5)	0.040 (4)	−0.015 (3)	0.004 (3)	−0.006 (3)
C63	0.040 (4)	0.102 (7)	0.068 (5)	−0.012 (4)	−0.006 (3)	−0.019 (5)
C64	0.060 (5)	0.074 (6)	0.140 (10)	0.014 (5)	−0.051 (6)	−0.018 (6)
C65	0.093 (7)	0.055 (5)	0.164 (13)	−0.006 (5)	−0.067 (8)	0.005 (6)
C66	0.053 (4)	0.045 (4)	0.140 (9)	−0.020 (3)	−0.030 (5)	0.007 (5)
Ir3A	0.076 (3)	0.0363 (19)	0.081 (3)	0.012 (2)	0.045 (4)	0.030 (2)
C52A	0.031 (4)	0.018 (3)	0.043 (4)	−0.001 (3)	0.007 (3)	0.003 (3)
C56A	0.046 (10)	0.032 (11)	0.034 (11)	−0.001 (7)	0.014 (7)	0.003 (7)
C45A	0.05 (2)	0.044 (17)	0.02 (3)	−0.015 (17)	0.006 (19)	−0.006 (17)
C54A	0.028 (4)	0.042 (4)	0.084 (6)	−0.003 (3)	0.009 (4)	0.014 (4)
C46A	0.033 (13)	0.027 (8)	0.038 (12)	0.001 (8)	0.006 (10)	−0.006 (8)
C58A	0.081 (19)	0.045 (10)	0.046 (9)	−0.011 (12)	−0.009 (11)	−0.006 (8)
C47A	0.031 (13)	0.039 (9)	0.033 (10)	−0.006 (9)	0.004 (9)	−0.004 (8)
C53A	0.028 (3)	0.021 (3)	0.049 (4)	0.005 (3)	−0.003 (3)	0.010 (3)
C48A	0.026 (11)	0.037 (10)	0.038 (10)	0.000 (8)	0.005 (8)	0.001 (7)
C55A	0.048 (11)	0.053 (16)	0.049 (16)	0.007 (11)	0.013 (10)	0.002 (12)
C49A	0.023 (10)	0.038 (11)	0.042 (12)	0.000 (7)	0.007 (8)	−0.001 (10)
C57A	0.052 (12)	0.038 (9)	0.029 (9)	−0.001 (9)	0.008 (8)	0.000 (7)
C50A	0.037 (10)	0.023 (10)	0.033 (12)	0.001 (7)	0.003 (8)	−0.013 (10)
C59A	0.064 (6)	0.031 (4)	0.053 (4)	−0.001 (4)	−0.011 (4)	−0.006 (3)
C51A	0.033 (11)	0.017 (9)	0.051 (12)	0.008 (7)	−0.005 (9)	0.003 (8)

### (η2:η2-Cycloocta-1,5-diene)(η6-toluene)iridium(I) tri-µ-chlorido-bis({3-[(di-tert-butylphosphanyl)oxy]-9,10-dioxoanthracen-2-yl}hydridoiridium(III)) toluene monosolvate, (1) Geometric parameters (Å, º)

**Table:**

Ir1—H1A	1.5498	C43—H43B	0.9800
Ir1—Cl1	2.4782 (11)	C43—H43C	0.9800
Ir1—Cl2	2.4818 (11)	C44—H44A	0.9800
Ir1—Cl3	2.5819 (11)	C44—H44B	0.9800
Ir1—P1	2.1819 (11)	C44—H44C	0.9800
Ir1—C2	2.001 (5)	Ir3—C46	2.413 (7)
Ir2—H2A	1.5502	Ir3—C47	2.361 (7)
Ir2—Cl1	2.5476 (11)	Ir3—C48	2.243 (6)
Ir2—Cl2	2.4963 (11)	Ir3—C49	2.317 (6)
Ir2—Cl3	2.4780 (12)	Ir3—C50	2.326 (6)
Ir2—P2	2.1899 (12)	Ir3—C51	2.250 (6)
Ir2—C24	2.008 (5)	Ir3—C52	2.150 (6)
P1—O3	1.651 (3)	Ir3—C53	2.139 (5)
P1—C15	1.879 (5)	Ir3—C56	2.129 (6)
P1—C19	1.885 (5)	Ir3—C57	2.142 (7)
P2—O6	1.656 (4)	C45—H45A	0.9800
P2—C37	1.867 (5)	C45—H45B	0.9800
P2—C41	1.874 (5)	C45—H45C	0.9800
O1—C9	1.221 (7)	C45—C46	1.491 (10)
O2—C10	1.221 (7)	C46—C47	1.393 (9)
O3—C3	1.398 (6)	C46—C51	1.433 (9)
O4—C31	1.219 (6)	C47—H47	1.0000
O5—C32	1.238 (6)	C47—C48	1.426 (9)
O6—C25	1.385 (6)	C48—H48	1.0000
C1—H1	0.9500	C48—C49	1.416 (9)
C1—C2	1.395 (7)	C49—H49	1.0000
C1—C11	1.395 (7)	C49—C50	1.387 (10)
C2—C3	1.421 (6)	C50—H50	1.0000
C3—C4	1.368 (7)	C50—C51	1.421 (9)
C4—H4	0.9500	C51—H51	1.0000
C4—C12	1.404 (7)	C52—H52	1.0000
C5—H5	0.9500	C52—C53	1.420 (9)
C5—C6	1.383 (8)	C52—C59	1.485 (10)
C5—C13	1.398 (8)	C53—H53	1.0000
C6—H6	0.9500	C53—C54	1.527 (10)
C6—C7	1.384 (9)	C54—H54A	0.9900
C7—H7	0.9500	C54—H54B	0.9900
C7—C8	1.399 (9)	C54—C55	1.504 (10)
C8—H8	0.9500	C55—H55A	0.9900
C8—C14	1.392 (8)	C55—H55B	0.9900
C9—C11	1.480 (7)	C55—C56	1.520 (10)
C9—C14	1.492 (8)	C56—H56	1.0000
C10—C12	1.464 (7)	C56—C57	1.429 (10)
C10—C13	1.490 (7)	C57—H57	1.0000
C11—C12	1.408 (7)	C57—C58	1.507 (11)
C13—C14	1.404 (8)	C58—H58A	0.9900
C15—C16	1.527 (8)	C58—H58B	0.9900
C15—C17	1.537 (7)	C58—C59	1.497 (11)
C15—C18	1.535 (7)	C59—H59A	0.9900
C16—H16A	0.9800	C59—H59B	0.9900
C16—H16B	0.9800	C60—H60A	0.9800
C16—H16C	0.9800	C60—H60B	0.9800
C17—H17A	0.9800	C60—H60C	0.9800
C17—H17B	0.9800	C60—C61	1.484 (11)
C17—H17C	0.9800	C61—C62	1.408 (11)
C18—H18A	0.9800	C61—C66	1.378 (13)
C18—H18B	0.9800	C62—H62	0.9500
C18—H18C	0.9800	C62—C63	1.362 (12)
C19—C20	1.530 (8)	C63—H63	0.9500
C19—C21	1.526 (7)	C63—C64	1.392 (16)
C19—C22	1.530 (7)	C64—H64	0.9500
C20—H20A	0.9800	C64—C65	1.424 (17)
C20—H20B	0.9800	C65—H65	0.9500
C20—H20C	0.9800	C65—C66	1.372 (13)
C21—H21A	0.9800	C66—H66	0.9500
C21—H21B	0.9800	Ir3A—C52A	2.16 (2)
C21—H21C	0.9800	Ir3A—C56A	2.08 (2)
C22—H22A	0.9800	Ir3A—C46A	2.41 (2)
C22—H22B	0.9800	Ir3A—C47A	2.38 (2)
C22—H22C	0.9800	Ir3A—C53A	2.13 (2)
C23—H23	0.9500	Ir3A—C48A	2.28 (2)
C23—C24	1.381 (8)	Ir3A—C49A	2.38 (2)
C23—C33	1.397 (7)	Ir3A—C57A	2.12 (2)
C24—C25	1.425 (6)	Ir3A—C50A	2.37 (2)
C25—C26	1.374 (7)	Ir3A—C51A	2.25 (2)
C26—H26	0.9500	C52A—H52A	1.0000
C26—C34	1.393 (8)	C52A—C53A	1.41 (3)
C27—H27	0.9500	C52A—C59A	1.48 (2)
C27—C28	1.372 (9)	C56A—H56A	1.0000
C27—C35	1.396 (7)	C56A—C55A	1.53 (2)
C28—H28	0.9500	C56A—C57A	1.44 (3)
C28—C29	1.397 (8)	C45A—H45D	0.9800
C29—H29	0.9500	C45A—H45E	0.9800
C29—C30	1.389 (8)	C45A—H45F	0.9800
C30—H30	0.9500	C45A—C46A	1.48 (4)
C30—C36	1.389 (8)	C54A—H54C	0.9900
C31—C33	1.486 (7)	C54A—H54D	0.9900
C31—C36	1.475 (7)	C54A—C53A	1.53 (2)
C32—C34	1.468 (7)	C54A—C55A	1.50 (2)
C32—C35	1.489 (8)	C46A—C47A	1.41 (3)
C33—C34	1.408 (6)	C46A—C51A	1.44 (3)
C35—C36	1.406 (7)	C58A—H58C	0.9900
C37—C38	1.534 (9)	C58A—H58D	0.9900
C37—C39	1.545 (8)	C58A—C57A	1.50 (2)
C37—C40	1.537 (8)	C58A—C59A	1.50 (2)
C38—H38A	0.9800	C47A—H47A	1.0000
C38—H38B	0.9800	C47A—C48A	1.41 (3)
C38—H38C	0.9800	C53A—H53A	1.0000
C39—H39A	0.9800	C48A—H48A	1.0000
C39—H39B	0.9800	C48A—C49A	1.42 (3)
C39—H39C	0.9800	C55A—H55C	0.9900
C40—H40A	0.9800	C55A—H55D	0.9900
C40—H40B	0.9800	C49A—H49A	1.0000
C40—H40C	0.9800	C49A—C50A	1.38 (3)
C41—C42	1.540 (8)	C57A—H57A	1.0000
C41—C43	1.536 (7)	C50A—H50A	1.0000
C41—C44	1.524 (7)	C50A—C51A	1.43 (3)
C42—H42A	0.9800	C59A—H59C	0.9900
C42—H42B	0.9800	C59A—H59D	0.9900
C42—H42C	0.9800	C51A—H51A	1.0000
C43—H43A	0.9800		
			
Cl1—Ir1—H1A	94.5	C56—Ir3—C51	99.1 (2)
Cl1—Ir1—Cl2	80.80 (4)	C56—Ir3—C52	94.3 (2)
Cl1—Ir1—Cl3	81.98 (4)	C56—Ir3—C53	80.5 (2)
Cl2—Ir1—H1A	82.5	C56—Ir3—C57	39.1 (3)
Cl2—Ir1—Cl3	78.35 (4)	C57—Ir3—C46	121.2 (3)
Cl3—Ir1—H1A	160.9	C57—Ir3—C47	154.4 (3)
P1—Ir1—H1A	91.8	C57—Ir3—C48	161.6 (3)
P1—Ir1—Cl1	101.24 (4)	C57—Ir3—C49	125.7 (3)
P1—Ir1—Cl2	174.13 (4)	C57—Ir3—C50	101.2 (3)
P1—Ir1—Cl3	107.33 (4)	C57—Ir3—C51	97.6 (3)
C2—Ir1—H1A	82.5	C57—Ir3—C52	80.3 (3)
C2—Ir1—Cl1	175.33 (14)	H45A—C45—H45B	109.5
C2—Ir1—Cl2	95.21 (13)	H45A—C45—H45C	109.5
C2—Ir1—Cl3	99.66 (14)	H45B—C45—H45C	109.5
C2—Ir1—P1	82.49 (13)	C46—C45—H45A	109.5
Cl1—Ir2—H2A	164.5	C46—C45—H45B	109.5
Cl2—Ir2—H2A	85.6	C46—C45—H45C	109.5
Cl2—Ir2—Cl1	79.18 (4)	C45—C46—Ir3	132.1 (5)
Cl3—Ir2—H2A	91.9	C47—C46—Ir3	71.0 (4)
Cl3—Ir2—Cl1	82.68 (4)	C47—C46—C45	123.0 (6)
Cl3—Ir2—Cl2	80.07 (4)	C47—C46—C51	117.8 (6)
P2—Ir2—H2A	88.6	C51—C46—Ir3	66.0 (4)
P2—Ir2—Cl1	106.79 (4)	C51—C46—C45	119.2 (6)
P2—Ir2—Cl2	173.06 (4)	Ir3—C47—H47	119.0
P2—Ir2—Cl3	103.93 (4)	C46—C47—Ir3	75.1 (4)
C24—Ir2—H2A	85.3	C46—C47—H47	119.0
C24—Ir2—Cl1	98.54 (13)	C46—C47—C48	120.7 (6)
C24—Ir2—Cl2	93.93 (14)	C48—C47—Ir3	67.5 (4)
C24—Ir2—Cl3	173.57 (14)	C48—C47—H47	119.0
C24—Ir2—P2	81.82 (14)	Ir3—C48—H48	120.1
Ir1—Cl1—Ir2	83.38 (3)	C47—C48—Ir3	76.5 (4)
Ir1—Cl2—Ir2	84.37 (4)	C47—C48—H48	120.1
Ir2—Cl3—Ir1	82.67 (4)	C49—C48—Ir3	74.8 (4)
O3—P1—Ir1	105.38 (12)	C49—C48—C47	119.8 (6)
O3—P1—C15	100.8 (2)	C49—C48—H48	120.1
O3—P1—C19	101.1 (2)	Ir3—C49—H49	119.7
C15—P1—Ir1	118.60 (17)	C48—C49—Ir3	69.1 (3)
C15—P1—C19	110.6 (2)	C48—C49—H49	119.7
C19—P1—Ir1	117.04 (16)	C50—C49—Ir3	73.0 (4)
O6—P2—Ir2	104.83 (13)	C50—C49—C48	119.4 (6)
O6—P2—C37	100.7 (2)	C50—C49—H49	119.7
O6—P2—C41	99.9 (2)	Ir3—C50—H50	119.3
C37—P2—Ir2	117.47 (17)	C49—C50—Ir3	72.3 (3)
C37—P2—C41	111.1 (3)	C49—C50—H50	119.3
C41—P2—Ir2	118.91 (17)	C49—C50—C51	119.9 (6)
C3—O3—P1	114.4 (3)	C51—C50—Ir3	69.0 (3)
C25—O6—P2	113.7 (3)	C51—C50—H50	119.3
C2—C1—H1	118.6	Ir3—C51—H51	119.9
C11—C1—H1	118.6	C46—C51—Ir3	78.4 (3)
C11—C1—C2	122.7 (4)	C46—C51—H51	119.9
C1—C2—Ir1	126.5 (3)	C50—C51—Ir3	74.9 (3)
C1—C2—C3	115.0 (4)	C50—C51—C46	120.2 (6)
C3—C2—Ir1	118.4 (3)	C50—C51—H51	119.9
O3—C3—C2	117.6 (4)	Ir3—C52—H52	113.3
C4—C3—O3	118.1 (4)	C53—C52—Ir3	70.3 (3)
C4—C3—C2	124.3 (4)	C53—C52—H52	113.3
C3—C4—H4	120.6	C53—C52—C59	125.6 (6)
C3—C4—C12	118.9 (4)	C59—C52—Ir3	113.9 (4)
C12—C4—H4	120.6	C59—C52—H52	113.3
C6—C5—H5	120.1	Ir3—C53—H53	114.6
C6—C5—C13	119.7 (6)	C52—C53—Ir3	71.1 (3)
C13—C5—H5	120.1	C52—C53—H53	114.6
C5—C6—H6	119.5	C52—C53—C54	120.3 (7)
C5—C6—C7	121.1 (6)	C54—C53—Ir3	114.7 (4)
C7—C6—H6	119.5	C54—C53—H53	114.6
C6—C7—H7	120.2	C53—C54—H54A	108.8
C6—C7—C8	119.5 (5)	C53—C54—H54B	108.8
C8—C7—H7	120.2	H54A—C54—H54B	107.7
C7—C8—H8	119.9	C55—C54—C53	113.7 (6)
C14—C8—C7	120.2 (6)	C55—C54—H54A	108.8
C14—C8—H8	119.9	C55—C54—H54B	108.8
O1—C9—C11	122.2 (5)	C54—C55—H55A	108.7
O1—C9—C14	120.0 (5)	C54—C55—H55B	108.7
C11—C9—C14	117.7 (4)	C54—C55—C56	114.1 (6)
O2—C10—C12	121.7 (5)	H55A—C55—H55B	107.6
O2—C10—C13	120.6 (5)	C56—C55—H55A	108.7
C12—C10—C13	117.7 (5)	C56—C55—H55B	108.7
C1—C11—C9	119.5 (4)	Ir3—C56—H56	113.7
C1—C11—C12	119.7 (5)	C55—C56—Ir3	114.0 (4)
C12—C11—C9	120.7 (5)	C55—C56—H56	113.7
C4—C12—C10	119.4 (4)	C57—C56—Ir3	70.9 (4)
C4—C12—C11	119.3 (5)	C57—C56—C55	123.5 (7)
C11—C12—C10	121.3 (4)	C57—C56—H56	113.8
C5—C13—C10	119.5 (5)	Ir3—C57—H57	115.0
C5—C13—C14	119.8 (5)	C56—C57—Ir3	70.0 (4)
C14—C13—C10	120.7 (5)	C56—C57—H57	115.0
C8—C14—C9	119.5 (5)	C56—C57—C58	121.0 (7)
C8—C14—C13	119.6 (5)	C58—C57—Ir3	113.2 (5)
C13—C14—C9	120.8 (5)	C58—C57—H57	115.0
C16—C15—P1	109.2 (3)	C57—C58—H58A	108.3
C16—C15—C17	107.0 (4)	C57—C58—H58B	108.3
C16—C15—C18	108.0 (5)	H58A—C58—H58B	107.4
C17—C15—P1	112.6 (4)	C59—C58—C57	115.9 (7)
C18—C15—P1	108.7 (3)	C59—C58—H58A	108.3
C18—C15—C17	111.2 (4)	C59—C58—H58B	108.3
C15—C16—H16A	109.5	C52—C59—C58	113.3 (6)
C15—C16—H16B	109.5	C52—C59—H59A	108.9
C15—C16—H16C	109.5	C52—C59—H59B	108.9
H16A—C16—H16B	109.5	C58—C59—H59A	108.9
H16A—C16—H16C	109.5	C58—C59—H59B	108.9
H16B—C16—H16C	109.5	H59A—C59—H59B	107.7
C15—C17—H17A	109.5	H60A—C60—H60B	109.5
C15—C17—H17B	109.5	H60A—C60—H60C	109.5
C15—C17—H17C	109.5	H60B—C60—H60C	109.5
H17A—C17—H17B	109.5	C61—C60—H60A	109.5
H17A—C17—H17C	109.5	C61—C60—H60B	109.5
H17B—C17—H17C	109.5	C61—C60—H60C	109.5
C15—C18—H18A	109.5	C62—C61—C60	119.4 (7)
C15—C18—H18B	109.5	C66—C61—C60	121.9 (7)
C15—C18—H18C	109.5	C66—C61—C62	118.6 (7)
H18A—C18—H18B	109.5	C61—C62—H62	120.2
H18A—C18—H18C	109.5	C63—C62—C61	119.6 (8)
H18B—C18—H18C	109.5	C63—C62—H62	120.2
C20—C19—P1	104.6 (4)	C62—C63—H63	119.3
C20—C19—C22	110.2 (4)	C62—C63—C64	121.5 (8)
C21—C19—P1	113.7 (4)	C64—C63—H63	119.3
C21—C19—C20	107.8 (5)	C63—C64—H64	120.2
C21—C19—C22	109.9 (4)	C63—C64—C65	119.5 (8)
C22—C19—P1	110.5 (4)	C65—C64—H64	120.2
C19—C20—H20A	109.5	C64—C65—H65	121.3
C19—C20—H20B	109.5	C66—C65—C64	117.3 (11)
C19—C20—H20C	109.5	C66—C65—H65	121.3
H20A—C20—H20B	109.5	C61—C66—H66	118.4
H20A—C20—H20C	109.5	C65—C66—C61	123.3 (9)
H20B—C20—H20C	109.5	C65—C66—H66	118.4
C19—C21—H21A	109.5	C52A—Ir3A—C46A	156.3 (12)
C19—C21—H21B	109.5	C52A—Ir3A—C47A	122.0 (12)
C19—C21—H21C	109.5	C52A—Ir3A—C48A	95.4 (11)
H21A—C21—H21B	109.5	C52A—Ir3A—C49A	94.5 (11)
H21A—C21—H21C	109.5	C52A—Ir3A—C50A	117.9 (12)
H21B—C21—H21C	109.5	C52A—Ir3A—C51A	153.3 (13)
C19—C22—H22A	109.5	C56A—Ir3A—C52A	95.0 (10)
C19—C22—H22B	109.5	C56A—Ir3A—C46A	103.5 (12)
C19—C22—H22C	109.5	C56A—Ir3A—C47A	126.1 (13)
H22A—C22—H22B	109.5	C56A—Ir3A—C53A	81.7 (9)
H22A—C22—H22C	109.5	C56A—Ir3A—C48A	160.4 (15)
H22B—C22—H22C	109.5	C56A—Ir3A—C49A	158.8 (13)
C24—C23—H23	119.0	C56A—Ir3A—C57A	40.2 (10)
C24—C23—C33	122.1 (4)	C56A—Ir3A—C50A	126.0 (12)
C33—C23—H23	119.0	C56A—Ir3A—C51A	100.9 (11)
C23—C24—Ir2	126.0 (3)	C47A—Ir3A—C46A	34.3 (8)
C23—C24—C25	116.2 (5)	C53A—Ir3A—C52A	38.3 (9)
C25—C24—Ir2	117.8 (4)	C53A—Ir3A—C46A	129.8 (13)
O6—C25—C24	118.1 (4)	C53A—Ir3A—C47A	103.3 (12)
C26—C25—O6	118.6 (4)	C53A—Ir3A—C48A	96.5 (11)
C26—C25—C24	123.3 (5)	C53A—Ir3A—C49A	116.8 (12)
C25—C26—H26	120.5	C53A—Ir3A—C50A	149.6 (13)
C25—C26—C34	119.1 (4)	C53A—Ir3A—C51A	165.5 (14)
C34—C26—H26	120.5	C48A—Ir3A—C46A	62.7 (9)
C28—C27—H27	119.7	C48A—Ir3A—C47A	35.1 (8)
C28—C27—C35	120.6 (5)	C48A—Ir3A—C49A	35.5 (8)
C35—C27—H27	119.7	C48A—Ir3A—C50A	62.0 (9)
C27—C28—H28	119.8	C49A—Ir3A—C46A	73.3 (9)
C27—C28—C29	120.4 (5)	C49A—Ir3A—C47A	62.4 (9)
C29—C28—H28	119.8	C57A—Ir3A—C52A	80.7 (9)
C28—C29—H29	120.4	C57A—Ir3A—C46A	123.0 (12)
C30—C29—C28	119.2 (6)	C57A—Ir3A—C47A	157.1 (12)
C30—C29—H29	120.4	C57A—Ir3A—C53A	93.2 (10)
C29—C30—H30	119.4	C57A—Ir3A—C48A	158.8 (14)
C29—C30—C36	121.2 (5)	C57A—Ir3A—C49A	123.6 (13)
C36—C30—H30	119.4	C57A—Ir3A—C50A	101.2 (12)
O4—C31—C33	121.4 (5)	C57A—Ir3A—C51A	97.9 (11)
O4—C31—C36	120.9 (5)	C50A—Ir3A—C46A	61.9 (9)
C36—C31—C33	117.6 (4)	C50A—Ir3A—C47A	72.2 (9)
O5—C32—C34	121.6 (5)	C50A—Ir3A—C49A	33.7 (8)
O5—C32—C35	120.0 (5)	C51A—Ir3A—C46A	35.7 (8)
C34—C32—C35	118.4 (4)	C51A—Ir3A—C47A	63.6 (9)
C23—C33—C31	119.0 (4)	C51A—Ir3A—C48A	76.2 (9)
C23—C33—C34	119.9 (5)	C51A—Ir3A—C49A	63.9 (9)
C34—C33—C31	121.1 (5)	C51A—Ir3A—C50A	35.8 (8)
C26—C34—C32	119.9 (4)	Ir3A—C52A—H52A	112.5
C26—C34—C33	119.5 (5)	C53A—C52A—Ir3A	69.7 (11)
C33—C34—C32	120.6 (5)	C53A—C52A—H52A	112.5
C27—C35—C32	119.7 (5)	C53A—C52A—C59A	129 (3)
C27—C35—C36	119.7 (5)	C59A—C52A—Ir3A	112.4 (18)
C36—C35—C32	120.6 (4)	C59A—C52A—H52A	112.5
C30—C36—C31	120.0 (4)	Ir3A—C56A—H56A	114.6
C30—C36—C35	118.9 (5)	C55A—C56A—Ir3A	115.1 (16)
C35—C36—C31	121.1 (5)	C55A—C56A—H56A	114.6
C38—C37—P2	106.4 (4)	C57A—C56A—Ir3A	71.3 (11)
C38—C37—C39	109.4 (4)	C57A—C56A—H56A	114.6
C38—C37—C40	107.9 (5)	C57A—C56A—C55A	120 (3)
C39—C37—P2	109.9 (4)	H45D—C45A—H45E	109.5
C40—C37—P2	114.7 (4)	H45D—C45A—H45F	109.5
C40—C37—C39	108.4 (5)	H45E—C45A—H45F	109.5
C37—C38—H38A	109.5	C46A—C45A—H45D	109.5
C37—C38—H38B	109.5	C46A—C45A—H45E	109.5
C37—C38—H38C	109.5	C46A—C45A—H45F	109.5
H38A—C38—H38B	109.5	H54C—C54A—H54D	107.7
H38A—C38—H38C	109.5	C53A—C54A—H54C	108.8
H38B—C38—H38C	109.5	C53A—C54A—H54D	108.8
C37—C39—H39A	109.5	C55A—C54A—H54C	108.8
C37—C39—H39B	109.5	C55A—C54A—H54D	108.8
C37—C39—H39C	109.5	C55A—C54A—C53A	113.8 (19)
H39A—C39—H39B	109.5	C45A—C46A—Ir3A	134 (3)
H39A—C39—H39C	109.5	C47A—C46A—Ir3A	71.6 (11)
H39B—C39—H39C	109.5	C47A—C46A—C45A	122 (3)
C37—C40—H40A	109.5	C47A—C46A—C51A	118.0 (19)
C37—C40—H40B	109.5	C51A—C46A—Ir3A	66.0 (11)
C37—C40—H40C	109.5	C51A—C46A—C45A	120 (3)
H40A—C40—H40B	109.5	H58C—C58A—H58D	107.4
H40A—C40—H40C	109.5	C57A—C58A—H58C	108.3
H40B—C40—H40C	109.5	C57A—C58A—H58D	108.3
C42—C41—P2	109.0 (3)	C59A—C58A—H58C	108.3
C43—C41—P2	111.9 (4)	C59A—C58A—H58D	108.3
C43—C41—C42	111.7 (5)	C59A—C58A—C57A	116 (2)
C44—C41—P2	108.2 (4)	Ir3A—C47A—H47A	119.3
C44—C41—C42	108.3 (5)	C46A—C47A—Ir3A	74.1 (12)
C44—C41—C43	107.6 (4)	C46A—C47A—H47A	119.3
C41—C42—H42A	109.5	C48A—C47A—Ir3A	68.8 (11)
C41—C42—H42B	109.5	C48A—C47A—C46A	120 (2)
C41—C42—H42C	109.5	C48A—C47A—H47A	119.3
H42A—C42—H42B	109.5	Ir3A—C53A—H53A	114.2
H42A—C42—H42C	109.5	C52A—C53A—Ir3A	72.0 (11)
H42B—C42—H42C	109.5	C52A—C53A—C54A	122 (3)
C41—C43—H43A	109.5	C52A—C53A—H53A	114.2
C41—C43—H43B	109.5	C54A—C53A—Ir3A	113.9 (15)
C41—C43—H43C	109.5	C54A—C53A—H53A	114.2
H43A—C43—H43B	109.5	Ir3A—C48A—H48A	119.5
H43A—C43—H43C	109.5	C47A—C48A—Ir3A	76.1 (13)
H43B—C43—H43C	109.5	C47A—C48A—H48A	119.5
C41—C44—H44A	109.5	C47A—C48A—C49A	121 (2)
C41—C44—H44B	109.5	C49A—C48A—Ir3A	75.9 (12)
C41—C44—H44C	109.5	C49A—C48A—H48A	119.5
H44A—C44—H44B	109.5	C56A—C55A—H55C	108.7
H44A—C44—H44C	109.5	C56A—C55A—H55D	108.7
H44B—C44—H44C	109.5	C54A—C55A—C56A	114.4 (19)
C47—Ir3—C46	33.9 (2)	C54A—C55A—H55C	108.7
C48—Ir3—C46	63.4 (2)	C54A—C55A—H55D	108.7
C48—Ir3—C47	36.0 (2)	H55C—C55A—H55D	107.6
C48—Ir3—C49	36.1 (2)	Ir3A—C49A—H49A	120.6
C48—Ir3—C50	63.9 (2)	C48A—C49A—Ir3A	68.7 (11)
C48—Ir3—C51	76.8 (2)	C48A—C49A—H49A	120.6
C49—Ir3—C46	74.0 (3)	C50A—C49A—Ir3A	73.0 (12)
C49—Ir3—C47	63.4 (2)	C50A—C49A—C48A	118 (2)
C49—Ir3—C50	34.8 (2)	C50A—C49A—H49A	120.6
C50—Ir3—C46	62.9 (2)	Ir3A—C57A—H57A	114.7
C50—Ir3—C47	73.8 (2)	C56A—C57A—Ir3A	68.4 (11)
C51—Ir3—C46	35.6 (2)	C56A—C57A—C58A	123 (4)
C51—Ir3—C47	63.3 (2)	C56A—C57A—H57A	114.7
C51—Ir3—C49	64.3 (2)	C58A—C57A—Ir3A	113.3 (16)
C51—Ir3—C50	36.1 (2)	C58A—C57A—H57A	114.7
C52—Ir3—C46	158.2 (2)	Ir3A—C50A—H50A	118.0
C52—Ir3—C47	124.3 (2)	C49A—C50A—Ir3A	73.3 (12)
C52—Ir3—C48	97.6 (2)	C49A—C50A—H50A	118.0
C52—Ir3—C49	97.1 (3)	C49A—C50A—C51A	121.7 (19)
C52—Ir3—C50	120.1 (2)	C51A—C50A—Ir3A	67.3 (11)
C52—Ir3—C51	155.6 (2)	C51A—C50A—H50A	118.0
C53—Ir3—C46	128.9 (2)	C52A—C59A—C58A	113 (2)
C53—Ir3—C47	103.7 (2)	C52A—C59A—H59C	108.9
C53—Ir3—C48	97.7 (2)	C52A—C59A—H59D	108.9
C53—Ir3—C49	119.3 (3)	C58A—C59A—H59C	108.9
C53—Ir3—C50	152.8 (3)	C58A—C59A—H59D	108.9
C53—Ir3—C51	164.3 (3)	H59C—C59A—H59D	107.7
C53—Ir3—C52	38.7 (2)	Ir3A—C51A—H51A	120.6
C53—Ir3—C57	91.9 (3)	C46A—C51A—Ir3A	78.3 (12)
C56—Ir3—C46	100.8 (3)	C46A—C51A—H51A	120.6
C56—Ir3—C47	123.2 (3)	C50A—C51A—Ir3A	76.9 (12)
C56—Ir3—C48	158.3 (3)	C50A—C51A—C46A	118 (2)
C56—Ir3—C49	158.5 (3)	C50A—C51A—H51A	120.6
C56—Ir3—C50	124.1 (3)		
			
Ir1—P1—O3—C3	−14.1 (4)	C29—C30—C36—C35	1.0 (8)
Ir1—P1—C15—C16	−31.9 (4)	C31—C33—C34—C26	176.5 (4)
Ir1—P1—C15—C17	−150.6 (3)	C31—C33—C34—C32	−6.4 (7)
Ir1—P1—C15—C18	85.7 (4)	C32—C35—C36—C30	179.2 (4)
Ir1—P1—C19—C20	61.8 (4)	C32—C35—C36—C31	−2.8 (7)
Ir1—P1—C19—C21	179.1 (3)	C33—C23—C24—Ir2	177.0 (4)
Ir1—P1—C19—C22	−56.8 (4)	C33—C23—C24—C25	0.2 (7)
Ir1—C2—C3—O3	−1.4 (6)	C33—C31—C36—C30	173.9 (4)
Ir1—C2—C3—C4	179.3 (4)	C33—C31—C36—C35	−4.1 (6)
Ir2—P2—O6—C25	−19.8 (3)	C34—C32—C35—C27	−174.3 (4)
Ir2—P2—C37—C38	59.7 (4)	C34—C32—C35—C36	5.3 (7)
Ir2—P2—C37—C39	−58.7 (5)	C35—C27—C28—C29	0.4 (8)
Ir2—P2—C37—C40	179.0 (4)	C35—C32—C34—C26	176.5 (4)
Ir2—P2—C41—C42	83.5 (4)	C35—C32—C34—C33	−0.6 (7)
Ir2—P2—C41—C43	−152.4 (3)	C36—C31—C33—C23	−173.8 (4)
Ir2—P2—C41—C44	−34.1 (4)	C36—C31—C33—C34	8.8 (7)
Ir2—C24—C25—O6	2.4 (6)	C37—P2—O6—C25	102.6 (3)
Ir2—C24—C25—C26	−178.1 (4)	C37—P2—C41—C42	−57.6 (4)
P1—O3—C3—C2	11.0 (6)	C37—P2—C41—C43	66.4 (4)
P1—O3—C3—C4	−169.7 (4)	C37—P2—C41—C44	−175.3 (4)
P2—O6—C25—C24	12.8 (5)	C41—P2—O6—C25	−143.4 (3)
P2—O6—C25—C26	−166.8 (4)	C41—P2—C37—C38	−158.5 (3)
O1—C9—C11—C1	−3.5 (9)	C41—P2—C37—C39	83.1 (4)
O1—C9—C11—C12	179.8 (6)	C41—P2—C37—C40	−39.3 (5)
O1—C9—C14—C8	7.0 (10)	Ir3—C46—C47—C48	−51.7 (6)
O1—C9—C14—C13	−175.4 (6)	Ir3—C46—C51—C50	64.7 (5)
O2—C10—C12—C4	10.3 (9)	Ir3—C47—C48—C49	−63.4 (5)
O2—C10—C12—C11	−168.4 (6)	Ir3—C48—C49—C50	−54.8 (6)
O2—C10—C13—C5	−9.2 (9)	Ir3—C49—C50—C51	−51.8 (6)
O2—C10—C13—C14	172.9 (6)	Ir3—C50—C51—C46	−66.5 (5)
O3—P1—C15—C16	82.4 (4)	Ir3—C52—C53—C54	−108.1 (5)
O3—P1—C15—C17	−36.3 (4)	Ir3—C52—C59—C58	21.4 (9)
O3—P1—C15—C18	−160.0 (4)	Ir3—C53—C54—C55	2.8 (10)
O3—P1—C19—C20	−52.0 (4)	Ir3—C56—C57—C58	−105.6 (6)
O3—P1—C19—C21	65.3 (4)	Ir3—C57—C58—C59	2.9 (11)
O3—P1—C19—C22	−170.6 (3)	C45—C46—C47—Ir3	−128.4 (7)
O3—C3—C4—C12	−176.0 (5)	C45—C46—C47—C48	179.9 (7)
O4—C31—C33—C23	7.5 (7)	C45—C46—C51—Ir3	126.3 (6)
O4—C31—C33—C34	−170.0 (5)	C45—C46—C51—C50	−169.1 (7)
O4—C31—C36—C30	−7.4 (7)	C46—C47—C48—Ir3	55.2 (6)
O4—C31—C36—C35	174.7 (5)	C46—C47—C48—C49	−8.2 (10)
O5—C32—C34—C26	−2.9 (7)	C47—C46—C51—Ir3	−50.4 (6)
O5—C32—C34—C33	179.9 (5)	C47—C46—C51—C50	14.2 (9)
O5—C32—C35—C27	5.2 (7)	C47—C48—C49—Ir3	64.3 (5)
O5—C32—C35—C36	−175.3 (4)	C47—C48—C49—C50	9.5 (10)
O6—P2—C37—C38	−53.4 (4)	C48—C49—C50—Ir3	53.0 (6)
O6—P2—C37—C39	−171.8 (4)	C48—C49—C50—C51	1.1 (10)
O6—P2—C37—C40	65.9 (5)	C49—C50—C51—Ir3	53.3 (6)
O6—P2—C41—C42	−163.3 (4)	C49—C50—C51—C46	−13.2 (10)
O6—P2—C41—C43	−39.3 (4)	C51—C46—C47—Ir3	48.1 (5)
O6—P2—C41—C44	79.1 (4)	C51—C46—C47—C48	−3.6 (10)
O6—C25—C26—C34	−179.7 (4)	C52—C53—C54—C55	84.4 (9)
C1—C2—C3—O3	174.9 (5)	C53—C52—C59—C58	−60.7 (10)
C1—C2—C3—C4	−4.4 (8)	C53—C54—C55—C56	−14.4 (12)
C1—C11—C12—C4	−3.2 (8)	C54—C55—C56—Ir3	19.7 (10)
C1—C11—C12—C10	175.6 (5)	C54—C55—C56—C57	−62.6 (10)
C2—C1—C11—C9	−174.9 (5)	C55—C56—C57—Ir3	106.7 (6)
C2—C1—C11—C12	1.9 (9)	C55—C56—C57—C58	1.1 (10)
C2—C3—C4—C12	3.3 (8)	C56—C57—C58—C59	82.7 (10)
C3—C4—C12—C10	−178.1 (5)	C57—C58—C59—C52	−15.9 (12)
C3—C4—C12—C11	0.7 (8)	C59—C52—C53—Ir3	105.8 (6)
C5—C6—C7—C8	0.4 (11)	C59—C52—C53—C54	−2.4 (9)
C5—C13—C14—C8	−1.5 (10)	C60—C61—C62—C63	−178.2 (7)
C5—C13—C14—C9	−179.1 (6)	C60—C61—C66—C65	179.0 (10)
C6—C5—C13—C10	−176.5 (6)	C61—C62—C63—C64	−2.0 (14)
C6—C5—C13—C14	1.4 (10)	C62—C61—C66—C65	−4.6 (16)
C6—C7—C8—C14	−0.5 (10)	C62—C63—C64—C65	−2.2 (17)
C7—C8—C14—C9	178.6 (6)	C63—C64—C65—C66	3.0 (19)
C7—C8—C14—C13	1.0 (10)	C64—C65—C66—C61	0.4 (19)
C9—C11—C12—C4	173.6 (5)	C66—C61—C62—C63	5.3 (12)
C9—C11—C12—C10	−7.7 (9)	Ir3A—C52A—C53A—C54A	−108 (2)
C10—C13—C14—C8	176.4 (6)	Ir3A—C52A—C59A—C58A	24 (7)
C10—C13—C14—C9	−1.2 (9)	Ir3A—C56A—C55A—C54A	10 (7)
C11—C1—C2—Ir1	177.7 (4)	Ir3A—C56A—C57A—C58A	−105 (2)
C11—C1—C2—C3	1.8 (8)	Ir3A—C46A—C47A—C48A	−53 (2)
C11—C9—C14—C8	−171.8 (6)	Ir3A—C46A—C51A—C50A	67.9 (18)
C11—C9—C14—C13	5.7 (9)	Ir3A—C47A—C48A—C49A	−63.7 (19)
C12—C10—C13—C5	170.3 (6)	Ir3A—C48A—C49A—C50A	−56 (2)
C12—C10—C13—C14	−7.6 (9)	Ir3A—C49A—C50A—C51A	−49 (2)
C13—C5—C6—C7	−0.9 (11)	Ir3A—C50A—C51A—C46A	−68.7 (18)
C13—C10—C12—C4	−169.1 (5)	C45A—C46A—C47A—Ir3A	−131 (4)
C13—C10—C12—C11	12.1 (8)	C45A—C46A—C47A—C48A	176 (4)
C14—C9—C11—C1	175.4 (5)	C45A—C46A—C51A—Ir3A	129 (4)
C14—C9—C11—C12	−1.3 (9)	C45A—C46A—C51A—C50A	−163 (4)
C15—P1—O3—C3	−138.0 (4)	C46A—C47A—C48A—Ir3A	55 (2)
C15—P1—C19—C20	−158.1 (3)	C46A—C47A—C48A—C49A	−8 (4)
C15—P1—C19—C21	−40.8 (5)	C47A—C46A—C51A—Ir3A	−51 (2)
C15—P1—C19—C22	83.3 (4)	C47A—C46A—C51A—C50A	17 (3)
C19—P1—O3—C3	108.2 (4)	C47A—C48A—C49A—Ir3A	64 (2)
C19—P1—C15—C16	−171.3 (3)	C47A—C48A—C49A—C50A	8 (3)
C19—P1—C15—C17	70.0 (4)	C53A—C52A—C59A—C58A	−58 (8)
C19—P1—C15—C18	−53.7 (4)	C53A—C54A—C55A—C56A	−3 (9)
C23—C24—C25—O6	179.5 (4)	C48A—C49A—C50A—Ir3A	54 (2)
C23—C24—C25—C26	−0.9 (7)	C48A—C49A—C50A—C51A	5 (4)
C23—C33—C34—C26	−1.0 (7)	C55A—C56A—C57A—Ir3A	109 (2)
C23—C33—C34—C32	176.1 (4)	C55A—C56A—C57A—C58A	4 (3)
C24—C23—C33—C31	−176.8 (4)	C55A—C54A—C53A—Ir3A	−5 (8)
C24—C23—C33—C34	0.8 (7)	C55A—C54A—C53A—C52A	78 (7)
C24—C25—C26—C34	0.7 (7)	C49A—C50A—C51A—Ir3A	51 (2)
C25—C26—C34—C32	−176.9 (4)	C49A—C50A—C51A—C46A	−17 (3)
C25—C26—C34—C33	0.3 (7)	C57A—C56A—C55A—C54A	−72 (7)
C27—C28—C29—C30	−0.7 (8)	C57A—C58A—C59A—C52A	−17 (10)
C27—C35—C36—C30	−1.2 (7)	C59A—C52A—C53A—Ir3A	103 (3)
C27—C35—C36—C31	176.8 (4)	C59A—C52A—C53A—C54A	−5 (4)
C28—C27—C35—C32	−179.9 (5)	C59A—C58A—C57A—Ir3A	3 (8)
C28—C27—C35—C36	0.5 (7)	C59A—C58A—C57A—C56A	81 (7)
C28—C29—C30—C36	−0.1 (8)	C51A—C46A—C47A—Ir3A	49 (2)
C29—C30—C36—C31	−177.0 (5)	C51A—C46A—C47A—C48A	−4 (4)

### (η2:η2-Cycloocta-1,5-diene)(η6-toluene)iridium(I) tri-µ-chlorido-bis({3-[(di-tert-butylphosphanyl)oxy]-9,10-dioxoanthracen-2-yl}hydridoiridium(III)) toluene monosolvate, (1) Hydrogen-bond geometry (Å, º)

**Table:**

*D*—H···*A*	*D*—H	H···*A*	*D*···*A*	*D*—H···*A*
C1—H1···Cl2	0.95	2.72	3.348 (5)	124
C20—H20*A*···Cl3	0.98	2.67	3.555 (5)	151
C22—H22*C*···Cl3	0.98	2.74	3.613 (6)	149
C23—H23···Cl2	0.95	2.70	3.316 (5)	123
C38—H38*A*···Cl1	0.98	2.71	3.589 (6)	149
C39—H39*C*···Cl1	0.98	2.66	3.559 (7)	152
C7—H7···O5^i^	0.95	2.44	3.262 (8)	145
C16—H16*A*···O5^ii^	0.98	2.40	3.306 (6)	154
C45—H45*A*···O1^i^	0.98	2.51	3.468 (11)	167
C45—H45*C*···O2^iii^	0.98	2.34	3.197 (11)	145
C48—H48···O4^i^	1.00	2.50	3.125 (8)	120
C49—H49···O4^i^	1.00	2.52	3.142 (9)	120
C52—H52···O4^i^	1.00	2.36	3.303 (8)	157

Symmetry codes: (i) −*x*+1, −*y*+1, −*z*+1; (ii) *x*−1, *y*, *z*; (iii) *x*+1, *y*, *z*.

### Di-µ-chlorido-bis(carbonyl{3-[(di-tert-butylphosphanyl)oxy]-9,10-dioxoanthracen-2-yl}hydridoiridium(I)) (2) Crystal data

**Table:**

[Ir_2_H_2_(C_23_H_26_O_2_P)_2_Cl_2_(CO)_2_]	*Z* = 1
*M**_r_* = 1244.15	*F*(000) = 608
Triclinic, *P*1	*D*_x_ = 1.388 Mg m^−^^3^
*a* = 8.8215 (3) Å	Cu *K*α radiation, λ = 1.54184 Å
*b* = 12.1331 (4) Å	Cell parameters from 13538 reflections
*c* = 14.4895 (3) Å	θ = 3.1–77.0°
α = 81.5747 (19)°	µ = 10.16 mm^−^^1^
β = 84.576 (2)°	*T* = 173 K
γ = 76.465 (3)°	Needle, light yellow
*V* = 1488.57 (7) Å^3^	0.19 × 0.04 × 0.03 mm

### Di-µ-chlorido-bis(carbonyl{3-[(di-tert-butylphosphanyl)oxy]-9,10-dioxoanthracen-2-yl}hydridoiridium(I)) (2) Data collection

**Table:**

XtaLAB Synergy, Dualflex, HyPix diffractometer	6137 independent reflections
Radiation source: micro-focus sealed X-ray tube, PhotonJet (Cu) X-ray Source	5626 reflections with *I* > 2σ(*I*)
Mirror monochromator	*R*_int_ = 0.046
Detector resolution: 10.0000 pixels mm^-1^	θ_max_ = 77.7°, θ_min_ = 3.1°
ω scans	*h* = −10→11
Absorption correction: multi-scan (CrysAlisPro; Rigaku OD, 2023)	*k* = −15→15
*T*_min_ = 0.459, *T*_max_ = 1.000	*l* = −18→11
20510 measured reflections	

### Di-µ-chlorido-bis(carbonyl{3-[(di-tert-butylphosphanyl)oxy]-9,10-dioxoanthracen-2-yl}hydridoiridium(I)) (2) Refinement

**Table:**

Refinement on *F*^2^	Primary atom site location: dual
Least-squares matrix: full	Secondary atom site location: difference Fourier map
*R*[*F*^2^ > 2σ(*F*^2^)] = 0.027	Hydrogen site location: mixed
*wR*(*F*^2^) = 0.067	H-atom parameters constrained
*S* = 1.06	*w* = 1/[σ^2^(*F*_o_^2^) + (0.0333*P*)^2^ + 0.1077*P*] where *P* = (*F*_o_^2^ + 2*F*_c_^2^)/3
6137 reflections	(Δ/σ)_max_ = 0.003
277 parameters	Δρ_max_ = 0.99 e Å^−^^3^
0 restraints	Δρ_min_ = −1.03 e Å^−^^3^

### Di-µ-chlorido-bis(carbonyl{3-[(di-tert-butylphosphanyl)oxy]-9,10-dioxoanthracen-2-yl}hydridoiridium(I)) (2) Special details

**Table:**

Geometry. All esds (except the esd in the dihedral angle between two l.s. planes) are estimated using the full covariance matrix. The cell esds are taken into account individually in the estimation of esds in distances, angles and torsion angles; correlations between esds in cell parameters are only used when they are defined by crystal symmetry. An approximate (isotropic) treatment of cell esds is used for estimating esds involving l.s. planes.
Refinement. Reflection contributions from highly disordered solvent were fixed and added to the calculated structure factors using the SQUEEZE routine of program Platon (Spek, 2015), which determined there to be 184 electrons in 492 Å^3^ treated this way per unit cell. Because the exact identity and amount of solvent were unknown, no solvent was included in the atom list or molecular formula. Thus all calculated quantities that derive from the molecular formula (e.g., F(000), density, molecular weight, etc.) are known to be inaccurate.The hydrido ligand's position was based on a peak found in the difference Fourier map. Once located, it was given a riding model that preserved its angle relative to the other ligands, but with its Ir–H distance fixed at approximately 1.55 Å (based on an average obtained from the Cambridge Structural Database for six-coordinate Ir complexes; Groom *et al.*, 2016). Independent spectroscopic experiments confirm the presence of this ligand.

### Di-µ-chlorido-bis(carbonyl{3-[(di-tert-butylphosphanyl)oxy]-9,10-dioxoanthracen-2-yl}hydridoiridium(I)) (2) Fractional atomic coordinates and isotropic or equivalent isotropic displacement parameters (Å^2^)

**Table:**

	*x*	*y*	*z*	*U*_iso_*/*U*_eq_	
Ir1	0.48460 (2)	0.63470 (2)	0.55302 (2)	0.02185 (6)	
H1A	0.336844	0.727771	0.574795	0.044*	
Cl1	0.32114 (8)	0.55180 (7)	0.46723 (5)	0.02494 (15)	
P1	0.61658 (10)	0.70682 (7)	0.64763 (5)	0.02345 (16)	
O1	0.0183 (3)	0.4082 (3)	0.73070 (17)	0.0457 (8)	
O2	0.3803 (3)	0.3658 (3)	1.01700 (16)	0.0363 (6)	
O3	0.5734 (4)	0.7724 (3)	0.37031 (18)	0.0497 (8)	
C1	0.2859 (4)	0.5016 (3)	0.6961 (2)	0.0260 (7)	
H1	0.223718	0.505372	0.645139	0.031*	
C2	0.4178 (4)	0.5481 (3)	0.6806 (2)	0.0237 (6)	
C3	0.5103 (4)	0.5376 (3)	0.7576 (2)	0.0223 (6)	
C4	0.4697 (4)	0.4833 (3)	0.8446 (2)	0.0233 (6)	
H4	0.534134	0.476328	0.894991	0.028*	
C5	0.0756 (5)	0.3309 (4)	1.0592 (2)	0.0374 (9)	
H5	0.140147	0.321387	1.109978	0.045*	
C6	−0.0738 (5)	0.3123 (4)	1.0755 (2)	0.0419 (10)	
H6	−0.111593	0.290184	1.137368	0.050*	
C7	−0.1693 (5)	0.3257 (4)	1.0014 (3)	0.0390 (9)	
H7	−0.272684	0.314287	1.012753	0.047*	
C8	−0.1126 (5)	0.3559 (4)	0.9114 (2)	0.0339 (8)	
H8	−0.176718	0.363464	0.860638	0.041*	
C9	0.0940 (4)	0.4107 (3)	0.7965 (2)	0.0302 (7)	
C10	0.2921 (4)	0.3870 (3)	0.9533 (2)	0.0269 (7)	
C11	0.2420 (4)	0.4497 (3)	0.7835 (2)	0.0243 (6)	
C12	0.3369 (4)	0.4393 (3)	0.8589 (2)	0.0237 (6)	
C13	0.1323 (4)	0.3635 (3)	0.9687 (2)	0.0279 (7)	
C14	0.0372 (4)	0.3751 (3)	0.8943 (2)	0.0287 (7)	
C15	0.6506 (4)	0.5901 (3)	0.7446 (2)	0.0249 (6)	
H15A	0.745979	0.532112	0.729981	0.030*	
H15B	0.664720	0.619963	0.802497	0.030*	
C16	0.4960 (5)	0.8365 (3)	0.6999 (2)	0.0319 (7)	
C17	0.3595 (5)	0.7995 (3)	0.7610 (3)	0.0350 (8)	
H17A	0.300180	0.765709	0.723284	0.052*	
H17B	0.290820	0.866313	0.785190	0.052*	
H17C	0.400591	0.742966	0.813315	0.052*	
C18	0.5891 (6)	0.8839 (4)	0.7628 (3)	0.0435 (10)	
H18A	0.636523	0.822425	0.810120	0.065*	
H18B	0.518912	0.944836	0.793613	0.065*	
H18C	0.671283	0.914644	0.724694	0.065*	
C19	0.4265 (6)	0.9315 (4)	0.6231 (3)	0.0423 (9)	
H19A	0.511215	0.957389	0.583710	0.063*	
H19B	0.360430	0.995829	0.651968	0.063*	
H19C	0.363424	0.901764	0.584677	0.063*	
C20	0.8163 (4)	0.7304 (3)	0.6026 (2)	0.0316 (7)	
C21	0.9217 (5)	0.7243 (4)	0.6829 (3)	0.0400 (9)	
H21A	0.877403	0.787223	0.719756	0.060*	
H21B	1.026473	0.730713	0.656990	0.060*	
H21C	0.928330	0.651212	0.723108	0.060*	
C22	0.8028 (6)	0.8456 (4)	0.5398 (3)	0.0430 (9)	
H22A	0.729995	0.850908	0.491437	0.064*	
H22B	0.905755	0.851252	0.510210	0.064*	
H22C	0.764030	0.908112	0.577805	0.064*	
C23	0.8956 (4)	0.6342 (4)	0.5443 (2)	0.0355 (8)	
H23A	0.901853	0.560058	0.582909	0.053*	
H23B	1.001115	0.643141	0.522377	0.053*	
H23C	0.834414	0.638018	0.490403	0.053*	
C24	0.5430 (4)	0.7199 (4)	0.4371 (2)	0.0332 (8)	

### Di-µ-chlorido-bis(carbonyl{3-[(di-tert-butylphosphanyl)oxy]-9,10-dioxoanthracen-2-yl}hydridoiridium(I)) (2) Atomic displacement parameters (Å^2^)

**Table:**

	*U*^11^	*U*^22^	*U*^33^	*U*^12^	*U*^13^	*U*^23^
Ir1	0.02111 (8)	0.02909 (9)	0.01463 (7)	−0.00636 (5)	0.00080 (5)	−0.00046 (5)
Cl1	0.0202 (3)	0.0360 (4)	0.0183 (3)	−0.0056 (3)	−0.0005 (2)	−0.0042 (3)
P1	0.0244 (4)	0.0288 (4)	0.0181 (3)	−0.0098 (3)	0.0011 (3)	−0.0016 (3)
O1	0.0365 (15)	0.084 (2)	0.0244 (12)	−0.0330 (15)	−0.0053 (10)	0.0024 (12)
O2	0.0339 (14)	0.0569 (18)	0.0209 (11)	−0.0203 (12)	−0.0055 (9)	0.0050 (10)
O3	0.068 (2)	0.055 (2)	0.0246 (12)	−0.0256 (16)	0.0018 (12)	0.0141 (12)
C1	0.0242 (16)	0.0332 (18)	0.0192 (13)	−0.0057 (13)	−0.0004 (11)	−0.0011 (12)
C2	0.0226 (15)	0.0298 (17)	0.0164 (13)	−0.0041 (13)	0.0027 (11)	−0.0016 (11)
C3	0.0211 (15)	0.0235 (16)	0.0215 (13)	−0.0042 (12)	0.0024 (11)	−0.0037 (11)
C4	0.0224 (15)	0.0279 (17)	0.0199 (13)	−0.0063 (13)	−0.0011 (11)	−0.0029 (11)
C5	0.037 (2)	0.055 (3)	0.0223 (15)	−0.0201 (18)	0.0020 (14)	0.0003 (15)
C6	0.040 (2)	0.065 (3)	0.0235 (16)	−0.024 (2)	0.0077 (14)	−0.0008 (16)
C7	0.0305 (19)	0.051 (3)	0.0374 (19)	−0.0184 (18)	0.0051 (15)	−0.0023 (16)
C8	0.0321 (19)	0.040 (2)	0.0308 (17)	−0.0137 (16)	0.0004 (14)	−0.0022 (14)
C9	0.0271 (17)	0.039 (2)	0.0238 (15)	−0.0105 (15)	−0.0013 (12)	0.0019 (13)
C10	0.0286 (17)	0.0316 (18)	0.0205 (14)	−0.0095 (14)	0.0021 (12)	−0.0014 (12)
C11	0.0226 (15)	0.0313 (18)	0.0192 (13)	−0.0078 (13)	0.0013 (11)	−0.0031 (12)
C12	0.0239 (16)	0.0275 (17)	0.0183 (13)	−0.0056 (13)	0.0011 (11)	−0.0006 (11)
C13	0.0274 (17)	0.0336 (19)	0.0241 (15)	−0.0119 (14)	0.0049 (12)	−0.0038 (13)
C14	0.0311 (18)	0.0330 (19)	0.0227 (14)	−0.0112 (15)	0.0023 (12)	−0.0021 (12)
C15	0.0244 (16)	0.0325 (18)	0.0179 (13)	−0.0083 (13)	−0.0024 (11)	0.0004 (12)
C16	0.038 (2)	0.0333 (19)	0.0239 (15)	−0.0103 (16)	0.0035 (13)	−0.0029 (13)
C17	0.036 (2)	0.032 (2)	0.0333 (17)	−0.0040 (16)	0.0083 (14)	−0.0061 (14)
C18	0.054 (3)	0.043 (2)	0.0380 (19)	−0.015 (2)	0.0017 (17)	−0.0161 (17)
C19	0.053 (3)	0.032 (2)	0.0361 (19)	−0.0038 (18)	0.0034 (17)	0.0041 (15)
C20	0.0313 (18)	0.042 (2)	0.0249 (15)	−0.0185 (16)	0.0027 (13)	−0.0007 (13)
C21	0.037 (2)	0.057 (3)	0.0325 (18)	−0.0242 (19)	−0.0022 (15)	−0.0054 (16)
C22	0.049 (2)	0.044 (2)	0.0389 (19)	−0.026 (2)	0.0031 (17)	0.0045 (16)
C23	0.0230 (17)	0.055 (2)	0.0289 (16)	−0.0120 (16)	0.0046 (13)	−0.0062 (15)
C24	0.0305 (18)	0.041 (2)	0.0276 (16)	−0.0075 (16)	−0.0024 (13)	−0.0043 (15)

### Di-µ-chlorido-bis(carbonyl{3-[(di-tert-butylphosphanyl)oxy]-9,10-dioxoanthracen-2-yl}hydridoiridium(I)) (2) Geometric parameters (Å, º)

**Table:**

Ir1—H1A	1.5529	C10—C12	1.480 (4)
Ir1—Cl1^i^	2.5353 (8)	C10—C13	1.493 (5)
Ir1—Cl1	2.4537 (7)	C11—C12	1.411 (4)
Ir1—P1	2.2650 (8)	C13—C14	1.398 (5)
Ir1—C2	2.093 (3)	C15—H15A	0.9900
Ir1—C24	1.932 (4)	C15—H15B	0.9900
P1—C15	1.835 (3)	C16—C17	1.539 (5)
P1—C16	1.896 (4)	C16—C18	1.533 (6)
P1—C20	1.891 (4)	C16—C19	1.538 (5)
O1—C9	1.222 (4)	C17—H17A	0.9800
O2—C10	1.223 (4)	C17—H17B	0.9800
O3—C24	1.125 (5)	C17—H17C	0.9800
C1—H1	0.9500	C18—H18A	0.9800
C1—C2	1.394 (5)	C18—H18B	0.9800
C1—C11	1.393 (4)	C18—H18C	0.9800
C2—C3	1.417 (4)	C19—H19A	0.9800
C3—C4	1.390 (4)	C19—H19B	0.9800
C3—C15	1.504 (5)	C19—H19C	0.9800
C4—H4	0.9500	C20—C21	1.541 (5)
C4—C12	1.384 (5)	C20—C22	1.538 (6)
C5—H5	0.9500	C20—C23	1.536 (5)
C5—C6	1.382 (6)	C21—H21A	0.9800
C5—C13	1.394 (5)	C21—H21B	0.9800
C6—H6	0.9500	C21—H21C	0.9800
C6—C7	1.394 (6)	C22—H22A	0.9800
C7—H7	0.9500	C22—H22B	0.9800
C7—C8	1.381 (5)	C22—H22C	0.9800
C8—H8	0.9500	C23—H23A	0.9800
C8—C14	1.389 (5)	C23—H23B	0.9800
C9—C11	1.477 (5)	C23—H23C	0.9800
C9—C14	1.495 (5)		
			
Cl1—Ir1—H1A	88.0	C14—C13—C10	121.4 (3)
Cl1^i^—Ir1—H1A	165.1	C8—C14—C9	119.4 (3)
Cl1—Ir1—Cl1^i^	82.39 (3)	C8—C14—C13	119.9 (3)
P1—Ir1—H1A	88.7	C13—C14—C9	120.6 (3)
P1—Ir1—Cl1	173.11 (3)	P1—C15—H15A	110.2
P1—Ir1—Cl1^i^	99.58 (3)	P1—C15—H15B	110.2
C2—Ir1—H1A	84.0	C3—C15—P1	107.5 (2)
C2—Ir1—Cl1^i^	84.99 (9)	C3—C15—H15A	110.2
C2—Ir1—Cl1	91.80 (9)	C3—C15—H15B	110.2
C2—Ir1—P1	81.83 (10)	H15A—C15—H15B	108.5
C24—Ir1—H1A	94.2	C17—C16—P1	107.8 (3)
C24—Ir1—Cl1^i^	96.98 (12)	C18—C16—P1	112.7 (3)
C24—Ir1—Cl1	89.22 (11)	C18—C16—C17	107.5 (3)
C24—Ir1—P1	97.06 (11)	C18—C16—C19	109.7 (4)
C24—Ir1—C2	177.89 (14)	C19—C16—P1	111.1 (2)
Ir1—Cl1—Ir1^i^	97.61 (3)	C19—C16—C17	107.9 (3)
C15—P1—Ir1	101.12 (11)	C16—C17—H17A	109.5
C15—P1—C16	106.24 (15)	C16—C17—H17B	109.5
C15—P1—C20	105.84 (16)	C16—C17—H17C	109.5
C16—P1—Ir1	113.99 (12)	H17A—C17—H17B	109.5
C20—P1—Ir1	117.45 (11)	H17A—C17—H17C	109.5
C20—P1—C16	110.74 (17)	H17B—C17—H17C	109.5
C2—C1—H1	118.8	C16—C18—H18A	109.5
C11—C1—H1	118.8	C16—C18—H18B	109.5
C11—C1—C2	122.4 (3)	C16—C18—H18C	109.5
C1—C2—Ir1	124.6 (2)	H18A—C18—H18B	109.5
C1—C2—C3	117.2 (3)	H18A—C18—H18C	109.5
C3—C2—Ir1	118.3 (2)	H18B—C18—H18C	109.5
C2—C3—C15	118.8 (3)	C16—C19—H19A	109.5
C4—C3—C2	120.9 (3)	C16—C19—H19B	109.5
C4—C3—C15	120.3 (3)	C16—C19—H19C	109.5
C3—C4—H4	119.5	H19A—C19—H19B	109.5
C12—C4—C3	121.1 (3)	H19A—C19—H19C	109.5
C12—C4—H4	119.5	H19B—C19—H19C	109.5
C6—C5—H5	119.8	C21—C20—P1	111.8 (2)
C6—C5—C13	120.4 (3)	C22—C20—P1	110.9 (3)
C13—C5—H5	119.8	C22—C20—C21	110.0 (3)
C5—C6—H6	119.9	C23—C20—P1	108.2 (3)
C5—C6—C7	120.3 (3)	C23—C20—C21	107.2 (3)
C7—C6—H6	119.9	C23—C20—C22	108.6 (3)
C6—C7—H7	120.2	C20—C21—H21A	109.5
C8—C7—C6	119.6 (4)	C20—C21—H21B	109.5
C8—C7—H7	120.2	C20—C21—H21C	109.5
C7—C8—H8	119.7	H21A—C21—H21B	109.5
C7—C8—C14	120.6 (3)	H21A—C21—H21C	109.5
C14—C8—H8	119.7	H21B—C21—H21C	109.5
O1—C9—C11	122.2 (3)	C20—C22—H22A	109.5
O1—C9—C14	120.3 (3)	C20—C22—H22B	109.5
C11—C9—C14	117.4 (3)	C20—C22—H22C	109.5
O2—C10—C12	121.8 (3)	H22A—C22—H22B	109.5
O2—C10—C13	120.9 (3)	H22A—C22—H22C	109.5
C12—C10—C13	117.2 (3)	H22B—C22—H22C	109.5
C1—C11—C9	119.3 (3)	C20—C23—H23A	109.5
C1—C11—C12	119.3 (3)	C20—C23—H23B	109.5
C12—C11—C9	121.3 (3)	C20—C23—H23C	109.5
C4—C12—C10	120.0 (3)	H23A—C23—H23B	109.5
C4—C12—C11	119.1 (3)	H23A—C23—H23C	109.5
C11—C12—C10	120.8 (3)	H23B—C23—H23C	109.5
C5—C13—C10	119.4 (3)	O3—C24—Ir1	177.8 (4)
C5—C13—C14	119.2 (3)		
			
Ir1—P1—C15—C3	−35.1 (2)	C6—C7—C8—C14	−1.4 (7)
Ir1—P1—C16—C17	62.1 (3)	C7—C8—C14—C9	−178.3 (4)
Ir1—P1—C16—C18	−179.4 (2)	C7—C8—C14—C13	0.3 (6)
Ir1—P1—C16—C19	−55.9 (3)	C9—C11—C12—C4	−176.1 (3)
Ir1—P1—C20—C21	−153.4 (2)	C9—C11—C12—C10	1.0 (5)
Ir1—P1—C20—C22	83.5 (3)	C10—C13—C14—C8	−178.1 (4)
Ir1—P1—C20—C23	−35.6 (3)	C10—C13—C14—C9	0.5 (5)
Ir1—C2—C3—C4	178.5 (2)	C11—C1—C2—Ir1	−176.7 (3)
Ir1—C2—C3—C15	0.7 (4)	C11—C1—C2—C3	1.9 (5)
O1—C9—C11—C1	10.0 (6)	C11—C9—C14—C8	169.5 (3)
O1—C9—C11—C12	−172.4 (4)	C11—C9—C14—C13	−9.1 (5)
O1—C9—C14—C8	−9.8 (6)	C12—C10—C13—C5	−170.2 (4)
O1—C9—C14—C13	171.6 (4)	C12—C10—C13—C14	8.8 (5)
O2—C10—C12—C4	−10.9 (5)	C13—C5—C6—C7	0.1 (7)
O2—C10—C12—C11	171.9 (3)	C13—C10—C12—C4	167.5 (3)
O2—C10—C13—C5	8.2 (6)	C13—C10—C12—C11	−9.6 (5)
O2—C10—C13—C14	−172.7 (4)	C14—C9—C11—C1	−169.2 (3)
C1—C2—C3—C4	−0.2 (5)	C14—C9—C11—C12	8.3 (5)
C1—C2—C3—C15	−178.0 (3)	C15—P1—C16—C17	−48.4 (3)
C1—C11—C12—C4	1.5 (5)	C15—P1—C16—C18	70.1 (3)
C1—C11—C12—C10	178.6 (3)	C15—P1—C16—C19	−166.3 (3)
C2—C1—C11—C9	175.0 (3)	C15—P1—C20—C21	−41.5 (3)
C2—C1—C11—C12	−2.6 (5)	C15—P1—C20—C22	−164.6 (3)
C2—C3—C4—C12	−0.9 (5)	C15—P1—C20—C23	76.4 (3)
C2—C3—C15—P1	24.8 (4)	C15—C3—C4—C12	176.9 (3)
C3—C4—C12—C10	−177.0 (3)	C16—P1—C15—C3	84.2 (2)
C3—C4—C12—C11	0.2 (5)	C16—P1—C20—C21	73.2 (3)
C4—C3—C15—P1	−153.1 (3)	C16—P1—C20—C22	−49.9 (3)
C5—C6—C7—C8	1.2 (7)	C16—P1—C20—C23	−168.9 (2)
C5—C13—C14—C8	0.9 (6)	C20—P1—C15—C3	−158.1 (2)
C5—C13—C14—C9	179.6 (4)	C20—P1—C16—C17	−162.8 (2)
C6—C5—C13—C10	177.9 (4)	C20—P1—C16—C18	−44.4 (3)
C6—C5—C13—C14	−1.2 (6)	C20—P1—C16—C19	79.2 (3)

Symmetry code: (i) −*x*+1, −*y*+1, −*z*+1.

### Di-µ-chlorido-bis(carbonyl{3-[(di-tert-butylphosphanyl)oxy]-9,10-dioxoanthracen-2-yl}hydridoiridium(I)) (2) Hydrogen-bond geometry (Å, º)

**Table:**

*D*—H···*A*	*D*—H	H···*A*	*D*···*A*	*D*—H···*A*
C15—H15*A*···O1^ii^	0.99	2.52	3.477 (5)	163
C15—H15*B*···O2^iii^	0.99	2.63	3.548 (4)	154
C23—H23*A*···Cl1^i^	0.98	2.84	3.312 (4)	111
C23—H23*B*···Cl1^ii^	0.98	2.86	3.757 (4)	152

Symmetry codes: (i) −*x*+1, −*y*+1, −*z*+1; (ii) *x*+1, *y*, *z*; (iii) −*x*+1, −*y*+1, −*z*+2.

## References

[bb1] Aguilà, D., Escribano, E., Speed, S., Talancón, D., Yermán, L. & Alvarez, S. (2009). *Dalton Trans.* pp. 6610–6625. 10.1039/b904938j 19672506

[bb2] Albrecht, M. & van Koten, G. (2001). *Angew. Chem. Int. Ed.* **40**, 3750–3781. 11668533

[bb3] Alig, L., Fritz, M. & Schneider, S. (2019). *Chem. Rev.* **119**, 2681–2751. 10.1021/acs.chemrev.8b00555 30596420

[bb4] Allevi, M., Capitani, D., Ettorre, A. & Mura, P. (1998). *Inorg. Chim. Acta*, **282**, 17–24.

[bb5] Alvarez, S. (2015). *Chem. Rev.* **115**, 13447–13483. 10.1021/acs.chemrev.5b00537 26575868

[bb50] Bandera, D. Baldridge, K. K., Linden, A. L., Spingler, B. & Siegel, J. S. (2020). *CSD Communication* (refcode BUNXEQ). CCDC, Cambridge, England.

[bb6] Boom, M. E. van der & Milstein, D. (2003). *Chem. Rev.* **103**, 1759–1792. 10.1021/cr960118r 12744693

[bb7] Byrn, M. P., Curtis, C. J., Khan, S. I., Sawin, P. A., Tsurumi, R. & Strouse, C. E. (1990). *J. Am. Chem. Soc.* **112**, 1865–1874.

[bb8] Choi, J., MacArthur, A. H. R., Brookhart, M. & Goldman, A. S. (2011). *Chem. Rev.* **111**, 1761–1779. 10.1021/cr1003503 21391566

[bb9] Cremades, E., Echeverría, J. & Alvarez, S. (2010). *Chem. A Eur. J.* **16**, 10380–10396. 10.1002/chem.200903032 20645325

[bb10] Dahlenburg, L., Heinemann, F. W., Kramer, D. & Menzel, R. (2008). *Acta Cryst.* C**64**, m144–m146. 10.1107/S0108270108004198 18322328

[bb11] Dahlenburg, L., Menzel, R. & Heinemann, F. W. (2007). *Eur. J. Inorg. Chem.* pp. 4364–4374.

[bb12] Dolomanov, O. V., Bourhis, L. J., Gildea, R. J., Howard, J. A. K. & Puschmann, H. (2009). *J. Appl. Cryst.* **42**, 339–341.

[bb51] Dorta, R., Broggini, D., Kissner, R. & Togni, A. (2004). *Chem. Eur. J.* **10**, 4546–4555. 10.1002/chem.200306008 15378634

[bb13] Drover, M. W., Bowes, E. G., Love, J. A. & Schafer, L. L. (2017). *Organometallics*, **36**, 331–341.

[bb14] Fisher, S. P., McArthur, S. G., Tej, V., Lee, S. E., Chan, A. L., Banda, I., Gregory, A., Berkley, K., Tsay, C., Rheingold, A. L., Guisado-Barrios, G. & Lavallo, V. (2020). *J. Am. Chem. Soc.* **142**, 251–256. 10.1021/jacs.9b10234 31804820

[bb15] Goldberg, J. M., Wong, G. W., Brastow, K. E., Kaminsky, W., Goldberg, K. I. & Heinekey, D. M. (2015). *Organometallics*, **34**, 753–762.

[bb16] Göttker-Schnetmann, I., White, P. & Brookhart, M. (2004). *J. Am. Chem. Soc.* **126**, 1804–1811. 10.1021/ja0385235 14871112

[bb17] Groom, C. R., Bruno, I. J., Lightfoot, M. P. & Ward, S. C. (2016). *Acta Cryst.* B**72**, 171–179. 10.1107/S2052520616003954 PMC4822653 27048719

[bb18] Il’in, S. G., Chetkina, L. A. & Golder, G. A. (1975). *Kristallografiya*, **20**, 1051.

[bb52] Ishii, Y., Onaka, K., Hirakawa, H. & Shiramizu, K. (2002). *Chem. Commun.* pp. 1150–1151. 10.1039/b201992m 12122709

[bb19] Kanchiku, S., Suematsu, H., Matsumoto, K., Uchida, T. & Katsuki, T. (2007). *Angew. Chem. Int. Ed.* **46**, 3889–3891. 10.1002/anie.200604385 17431871

[bb20] Ketker, S. N., Kelley, M., Fink, M. & Ivey, R. C. (1981). *J. Mol. Struct.* **77**, 127–138.

[bb21] Lenstra, A. T. H. & van Loock, J. F. J. (1984). *Bull. Soc. Chim.* **93**, 1053–1055.

[bb53] Linden, A. L. & Dorta, R. (2020). *CSD Communication* (refcode VUQBUH). CCDC, Cambridge, England.

[bb22] Maekawa, M., Hashimoto, N., Sugimoto, K., Kuroda-Sowa, T., Suenaga, Y. & Munakata, M. (2003). *Inorg. Chim. Acta*, **344**, 143–157.

[bb23] Maekawa, M., Suenaga, Y., Kuroda-Sowa, T. & Munakata, M. (2004*a*). *Inorg. Chim. Acta*, **357**, 331–338.

[bb24] Maekawa, M., Suenaga, Y., Kuroda-Sowa, T. & Munakata, M. (2004*b*). *Anal. Sci. X*, **20**, X11–X12. 10.2116/analsci.17.1361 11759528

[bb54] Melcher, M., von Wachenfeldt, H., Sundin, A. & Strand, D. (2015). *Chem. Eur. J.* **21**, 531–535. 10.1002/chem.201405729 25413863

[bb25] Morales-Morales, D. (2008). *Mini-Rev. Org. Chem.* **5**, 141–152.

[bb26] Morales-Morales, D. (2018). Editor. *Pincer Compounds: Chemistry and Applications.* Cambridge, Massachusetts: Elsevier.

[bb47] Moulton, C. J. & Shaw, B. L. (1976). *J. Chem. Soc. Dalton Trans.*, pp. 1020–1024.

[bb27] Muetterties, E. L., Bleeke, J. R. & Sievert, A. C. (1979). *J. Organomet. Chem.* **178**, 197–216.

[bb55] Muldoon, J. & Brown, S. N. (2003). *Organometallics*, **22**, 4480–4489.

[bb28] Mura, P. (2000). *J. Coord. Chem.* **51**, 253–260.

[bb29] Pascal, R. A., Dudnikov, A., Love, L. A., Geng, X., Dougherty, K. J., Mague, J. T., Kraml, C. M. & Byrne, N. (2017). *Eur. J. Org. Chem.* pp. 4194–4200.

[bb30] Rigaku OD (2023). *CrysAlis PRO*. Rigaku Oxford Diffraction, Yarnton, England.

[bb31] Roddick, D. M. (2013). In *Topics in Organometallic Chemistry: Organometallic Pincer Chemistry*, Vol 40, edited by G. van Koten & D. Milstein, pp. 49–88. Berlin, Heidelberg: Springer.

[bb32] Shafiei-Haghighi, S., Singer, L. M., Tamang, S. R. & Findlater, M. (2018). *Polyhedron*, **143**, 126–131.

[bb33] Sheldrick, G. M. (2015*a*). *Acta Cryst.* A**71**, 3–8.

[bb34] Sheldrick, G. M. (2015*b*). *Acta Cryst.* C**71**, 3–8.

[bb35] Sievers, R., Sellin, M., Rupf, S. M., Parche, J. & Malischewski, M. (2022). *Angew. Chem. Int. Ed.* **61**, e202211147. 10.1002/anie.202211147 PMC9826324 35984742

[bb36] Sievert, A. C. & Muetterties, E. L. (1981). *Inorg. Chem.* **20**, 489–501.

[bb37] Spek, A. L. (2015). *Acta Cryst.* C**71**, 9–18. 10.1107/S2053229614024929 25567569

[bb38] Stiefel, E. I. & Brown, G. F. (1972). *Inorg. Chem.* **11**, 434–436.

[bb39] Sumitani, R., Kuwahara, D. & Mochida, T. (2023). *Inorg. Chem.* **62**, 2169–2180. 10.1021/acs.inorgchem.2c03865 PMC9907349 36701547

[bb40] Tatarin, S. V., Smirnov, D. E., Taydakov, I. V., Metlin, M. T., Emets, V. V. & Bezzubov, S. I. (2023). *Dalton Trans.* **52**, 6435–6450. 10.1039/d3dt00200d 37092600

[bb56] Tejel, C., Ciriano, M.., Passarelli, V., López, J.. & de Bruin, B. (2008). *Chem. Eur. J.* **14**, 10985–10998. 10.1002/chem.200801615 18979469

[bb41] Viciano, M., Poyatos, M., Sanaú, M., Peris, E., Rossin, A., Ujaque, G. & Lledós, A. (2006). *Organometallics*, **25**, 1120–1134.

[bb42] Ward, T. M., Schafer, O., Daul, C. & Hofmann, P. (1997). *Organometallics*, **16**, 3207–3215.

[bb43] Wilklow-Marnell, M. & Brennessel, W. W. (2019). *Polyhedron*, **160**, 83–91.

[bb44] Yellowlees, L., Elliot, M., Parsons, S. & Messenger, D. (2005). Private communication (refcode: MASNEA). CCDC, Cambridge, England.

[bb45] Yellowlees, L. J. & Macnamara, K. G. (2003). In *Comprehensive Coordination Chemistry II*, Vol. 6, edited by J. A. McCleverty & T. J. Meyer, pp. 147–246. Oxford: Elsevier.

[bb46] Zhang, X., Emge, T. J. & Goldman, A. S. (2004). *Inorg. Chim. Acta*, **357**, 3014–3018.

